# Cohesin-protein Shugoshin-1 controls cardiac automaticity via HCN4 pacemaker channel

**DOI:** 10.1038/s41467-021-22737-5

**Published:** 2021-05-05

**Authors:** Donghai Liu, Andrew Taehun Song, Xiaoyan Qi, Patrick Piet van Vliet, Jiening Xiao, Feng Xiong, Gregor Andelfinger, Stanley Nattel

**Affiliations:** 1 Montreal Heart Institute, Department of Medicine, Université de Montréal, Montréal, QC Canada; 2 Department of Anatomy and Cell Biology, McGill University, Montréal, QC Canada; 3 Cardiovascular Genetics, Department of Pediatrics, Centre Hospitalier Universitaire Sainte-Justine Research Centre, University of Montreal, Montréal, QC Canada; 4 LIA (International Associated Laboratory) INSERM, Marseille, France; 5 LIA (International Associated Laboratory) Centre Hospitalier Universitaire Sainte-Justine, Montréal, QC Canada; 6 Department of Pharmacology and Therapeutics, McGill University, Montréal, QC Canada; 7 Department of Pediatrics, University of Montreal, Montréal, QC Canada; 8 Department of Biochemistry, University of Montreal, Montréal, QC Canada; 9 Institute of Pharmacology, West German Heart and Vascular Center, Faculty of Medicine, University Duisburg-Essen, Essen, Germany; 10 IHU LIRYC Institute, Fondation Bordeaux Université, Bordeaux, France

**Keywords:** Cardiovascular models, Ion transport, Cardiovascular genetics

## Abstract

Endogenous cardiac pacemaker function regulates the rate and rhythm of cardiac contraction. The mutation p.Lys23Glu in the cohesin protein Shugoshin-1 causes severe heart arrhythmias due to sinoatrial node dysfunction and a debilitating gastrointestinal motility disorder, collectively termed the Chronic Atrial and Intestinal Dysrhythmia Syndrome, linking Shugoshin-1 and pacemaker activity. Hyperpolarization-activated, cyclic nucleotide-gated cation channel 4 (HCN4) is the predominant pacemaker ion-channel in the adult heart and carries the majority of the “funny” current, which strongly contributes to diastolic depolarization in pacemaker cells. Here, we study the mechanism by which Shugoshin-1 affects cardiac pacing activity with two cell models: neonatal rat ventricular myocytes and Chronic Atrial and Intestinal Dysrhythmia Syndrome patient-specific human induced pluripotent stem cell derived cardiomyocytes. We find that Shugoshin-1 interacts directly with HCN4 to promote and stabilize cardiac pacing. This interaction enhances funny-current by optimizing HCN4 cell-surface expression and function. The clinical p.Lys23Glu mutation leads to an impairment in the interaction between Shugoshin-1 and HCN4, along with depressed funny-current and dysrhythmic activity in induced pluripotent stem cell derived cardiomyocytes derived from Chronic Atrial and Intestinal Dysrhythmia Syndrome patients. Our work reveals a critical non-canonical, cohesin-independent role for Shugoshin-1 in maintaining cardiac automaticity and identifies potential therapeutic avenues for cardiac pacemaking disorders, in particular Chronic Atrial and Intestinal Dysrhythmia Syndrome.

## Abstract (web summary)

A mutation in Shugoshin-1 causes the Chronic Atrial and Intestinal Dysrhythmia (CAID) Syndrome, but the underlying mechanisms are unknown. Here, the authors show that Shugoshin-1 controls cardiac pacemaker activity by interacting with HCN4 to enhance its cell-surface expression, and that the CAID-Syndrome mutation disrupts cardiac pacemaking by interfering with this important non-canonical interaction.

## Introduction

Cohesins are ring-like structures consisting of four core units: two Structural Maintenance of Chromosomes protein subunits (SMC1 and SMC3); a kleisin subunit (RAD21); and a Stromal Antigen subunit (SA1 or SA2)^1^. During the G1 stage of mitosis, open cohesins are “loaded” onto chromatids via Nipped-B-Like Protein (NIPBL)/MAU2 heterodimers, upon which they close and encircle sister chromatids. Conversely, cohesins are progressively unloaded by Wings Apart-Like homolog WAPL and PDS5, with only the centromere cohesins being maintained until chromosomes are properly aligned^1,2^. To prevent premature unloading, cohesins are stabilized by Shugoshin 1 (SGO1), which recruits protein-phosphatase 2 A (PP2A) to antagonize SA-phosphorylation^3,4^. SGO1 is removed when phosphorylated by Polo-Like Kinase 1 (PLK1), which leads to cohesin cleavage by separase. Cohesins are also epigenetically involved in transcriptional regulation via Topologically Associating Domains (TADs) and enhancer-promoter loops via interactions with the insulator protein CTCF^4–6^. Mutations in cohesin core proteins, NIPBL, ESCO1, and ESCO2 acetyltransferases, or Histone Deacetylase 8 (HDAC8) have all been associated with “cohesinopathies”, in which syndrome-specific changes in cell-cycling and/or transcriptional regulation produce craniofacial, neural and cardiac defects, gastrointestinal dysfunction, developmental delay, or premature ageing^1,5,6^.

SGO1 function is necessary for correct cell division in mammals. In humans, SGO1 somatic mutations are found in colorectal cancers and are associated with chromosomal instability^7,8^. Depletion of SGO1 in HeLa cells results in similar anomalies: premature loss of centromeric cohesin, premature segregation of sister chromatids, and mitotic arrest^9^. In mice, knockout of both Sgo1 alleles results in embryonic lethality whereas Sgo1^+/−^ mice show a propensity for colon cancer while remaining viable and fertile^10^. Sgo1^+/−^ mice also show increases in β-amyloid accumulation (of unclear mechanism) in the brain, resembling those seen in Alzheimer’s disease^11^. We recently identified a series of patients presenting with a combination of heart-rhythm disorders, mainly very slow rhythms due to failure of endogenous cardiac pacemaking function requiring the implantation of artificial pacemakers, and chronic intestinal pseudo-obstruction (CIPO)^5^. We termed this syndrome, with evidence of both atrial and intestinal dysrhythmia, Chronic Atrial and Intestinal Dysrhythmia (CAID) syndrome. All CAID patients were homozygous for a recessive point mutation (A > G) in *SGO1*, leading to a lysine-to-glutamic acid change at the highly conserved amino acid 23 (p.Lys23Glut: SGO1-K23E). In contrast to other cohesinopathies, CAID-patients have no craniofacial or structural cardiac defects, nor premature ageing^12^. CAID-related symptoms appear to mainly, and postnatally, affect the heart and gut, suggesting that the SGO1-K23E mutation likely leads to a change of function with relatively mild, but cell-specific effects. To investigate the molecular mechanisms leading to CAID-related clinical manifestations, we recently performed multiomics studies on primary control versus patient-derived dermal fibroblasts, which pointed to canonical (i.e., cell cycling and epigenetic) as well as non-canonical changes in signaling pathways, including disturbed transforming growth-factor beta signaling and altered fibroblast ion-channel function^12^.

Here, we show that disordered cardiac pacemaker function in CAID syndrome is attributable to disruption by the SGO1-K23E mutation of an SGO1 non-canonical function to enhance the cardiac pacemaker funny current (I_f_).

## Results

### SGO1 promotes automaticity of NRVMs by enhancing I_f_

**Fig. 1:**
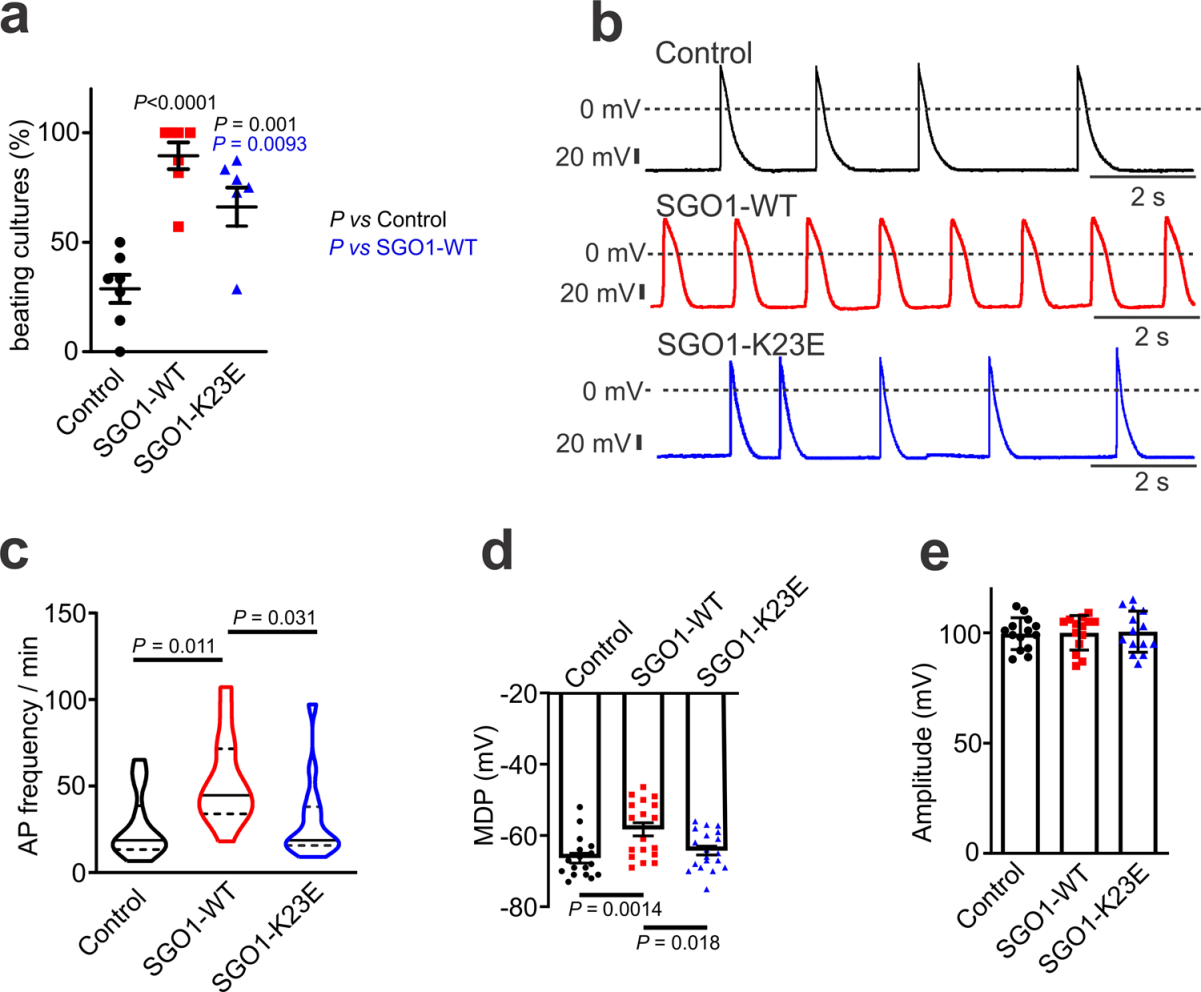
SGO1-WT promotes automaticity of NRVMs. **a** Transduction of SGO1-WT increased the number of spontaneously beating NRVM monolayers compared to control and SGO1-K23E transduced NRVMs after 30 to 48 h transduction; SGO1-K23E had weaker effects than WT on NRVMs. Each point in the plot represents the percentage of wells of a 24-well plate that were beating for each experiment. *n* = 7 biologically independent experiments with independent preparations on different days. Data are expressed as mean ± SEM. One-way ANOVA with Tukey’s multiple comparison test. **b** Representative action-potential (AP) recordings in NRVM monolayers of control (black), SGO1-WT (red) and SGO1-K23E (blue) transduced preparations. The short vertical lines at left are 20 mV scales. **c** SGO1-WT transduction promoted spontaneous AP firing in NRVMs compared to control and SGO1-K23E. *n* = 14 biologically independent cells per group. Data are represented as violin plots, with median (solid line) and interquartile range (dotted line). One-way ANOVA with Tukey’s multiple comparison test. **d** Reduction of MDP in SGO1-WT NRVMs. *n* = 17–19 biologically independent cells per group. Data are expressed as mean ± SEM. One-way ANOVA with Tukey’s multiple comparison test. **e** Effect of SGO1-WT and SGO1-K23E transduction on AP amplitude in NRVMs. *n* = 14 biologically independent cells/group. Data are expressed as mean ± SEM.

Cardiac automaticity depends on specialized cellular pacemaker function, as well as the structural integrity of cardiac pacemaker tissue. To determine whether SGO1 might play a direct role in cellular automaticity, we investigated the effects of ectopic expression of SGO1 and the mutated SGO1-K23E protein on the automaticity of cultured neonatal rat ventricular myocyte (NRVM) monolayers. Lentivirus was used to carry empty vector (control), wild type SGO1 (SGO1-WT) and K23E mutant SGO1 (SGO1-K23E). The numbers of spontaneous beating cultures were counted 30–48 h after lentiviral transduction. We found that SGO1-WT transduced NRVMs had a higher percentage of spontaneously beating cultures compared to control and SGO1-K23E transduced NRVMs (Fig. 1a). This result indicates that SGO1 enhances NRVM automaticity and that the SGO1-K23E mutation impairs this function. To further assess the effects of SGO1 on automaticity, we recorded spontaneous action potentials (APs) from NRVM monolayers transduced with SGO1 and SGO1-K23E with the perforated patch-clamp technique to maintain physiological intracellular contents. Robust spontaneous APs were recorded from these NRVMs (Fig. 1b). The spontaneous AP firing frequency in SGO1-WT transduced NRVMs (52.8 ± 6.8/min) was significantly increased compared with control (23.9 ± 6.4/min) and SGO1-K23E transduced (26.4 ± 5.0/min) NRVMs (Fig. 1c). SGO1 transduction also reduced maximum diastolic potential (MDP) in NRVM monolayers (Fig. 1d), pointing to either a reduced outward current or increased inward current. No difference in AP amplitude was observed among these groups (Fig. 1e).

**Fig. 2:**
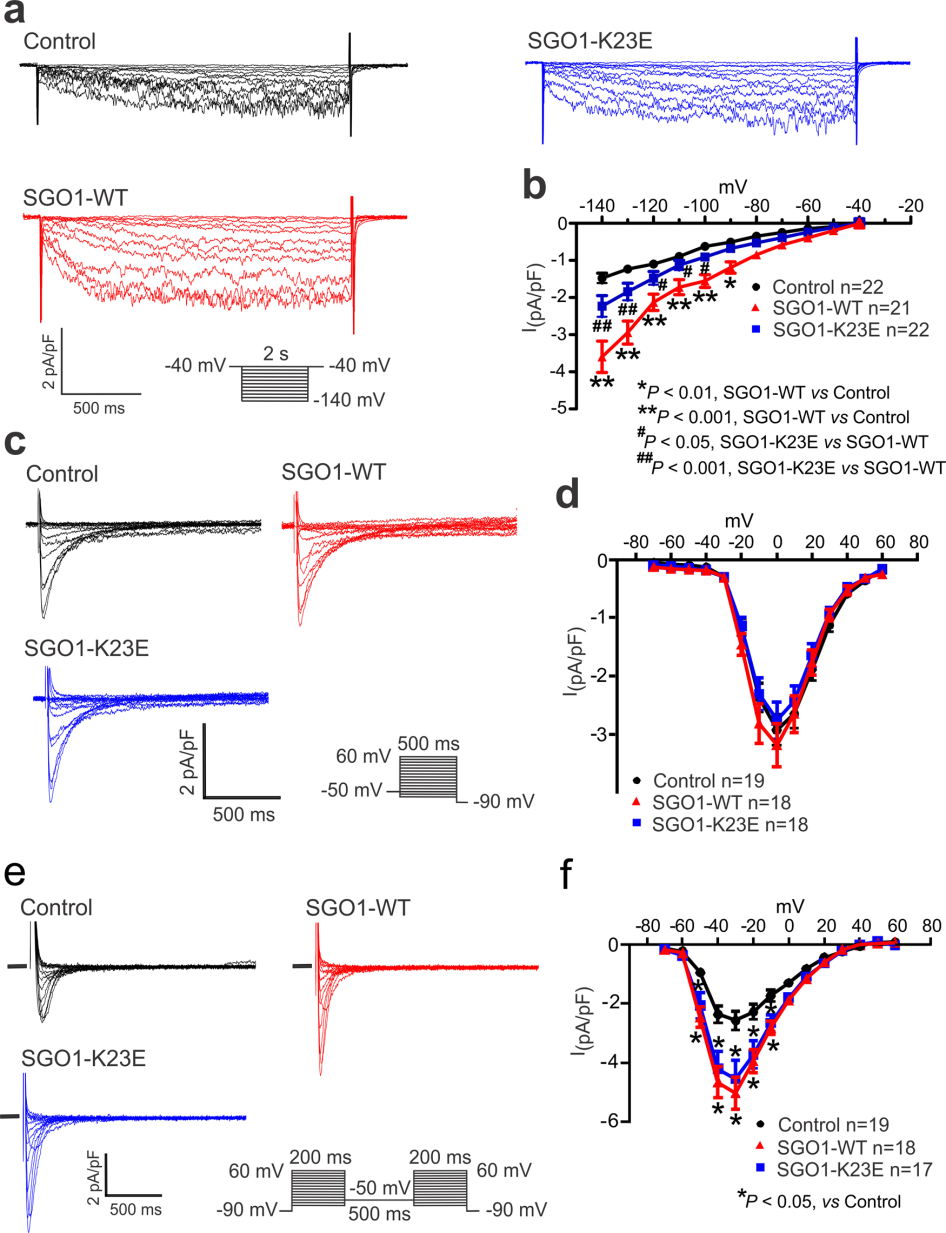
Effect of SGO1-WT and SGO1-K23E on I_f_, I_CaL_ and I_CaT_ in NRVMs. **a** Representative I_f_ recordings from NRVMs of control (black), SGO1-WT (red) and SGO1-K23E (Blue) preparations. **b** Average current density-voltage (I-V) relation of I_f_ for control, SGO1-WT and SGO1-K23E transduced NRVMs. I_f_ was elicited from a holding potential of −40 mV, using 2 s voltage steps from −140 mV to −40 mV in 10 mV increments with an interpulse interval of 10 s. Data are mean ± SEM. Two-way AVOVA. **c** Representative I_CaL_ recordings in control (black), SGO1-WT (red) and SGO1-K23E transfected (blue) NRVMs. **d** I-V relation of I_CaL_ plotted from control, SGO1-WT and SGO1-K23E NRVMs. Data are expressed as mean ± SEM. **e** I_CaT_ recordings in NRVMs. Two series of voltage steps of 200 ms from −90 mV to +60 mV were applied, separated by 500 ms intervals at −50 mV to inactive I_CaT_ (protocol delivered at 0.1 Hz). I_CaT_ was obtained by subtracting the currents with a holding potential of −50 mV (only I_CaL_) from those with a holding potential of −90 mV (I_CaL_ + I_CaT_). **f** I-V relation for I_CaT_. Data are mean ± SEM. Two-way AVOVA. n’s in figure are number of separate cells studied.

I_f_ acts as a pacemaker current in cardiac cells showing spontaneous automaticity, including cells in the principal pacemaker sinoatrial node region, carrying an inward current at negative potentials that initiates spontaneous diastolic depolarization, contributing to the “voltage clock” mechanism of cardiac automaticity^13^. We therefore considered whether transduction of SGO1 or its K23E mutant affects I_f_ in NRVMs. Typical I_f_ recordings were obtained from NRVMs, similar to previously reported results^14^ (Fig. 2a). The I_f_ current density was significantly increased by transduction with SGO1-WT (Fig. 2b), whereas SGO1-K23E transduced cells showed a greatly attenuated effect (Fig. 2b). In addition to I_f_, T-type (I_CaT_) and L-type (I_CaL_) calcium currents are involved in diastolic depolarization and the subsequent AP upstroke^15^. I_CaL_ recording showed no differences among control, SGO1-WT and SGO1-K23E transduced NRVMs (Fig. 2c, d). I_CaT_ was enhanced by transduction with SGO1-WT; however, SGO1-K23E transduction had a similar effect (Fig. 2e, f), making I_CaT_ an unlikely mediator for the SGO1 effect on automaticity in CAID.

**Fig. 3:**
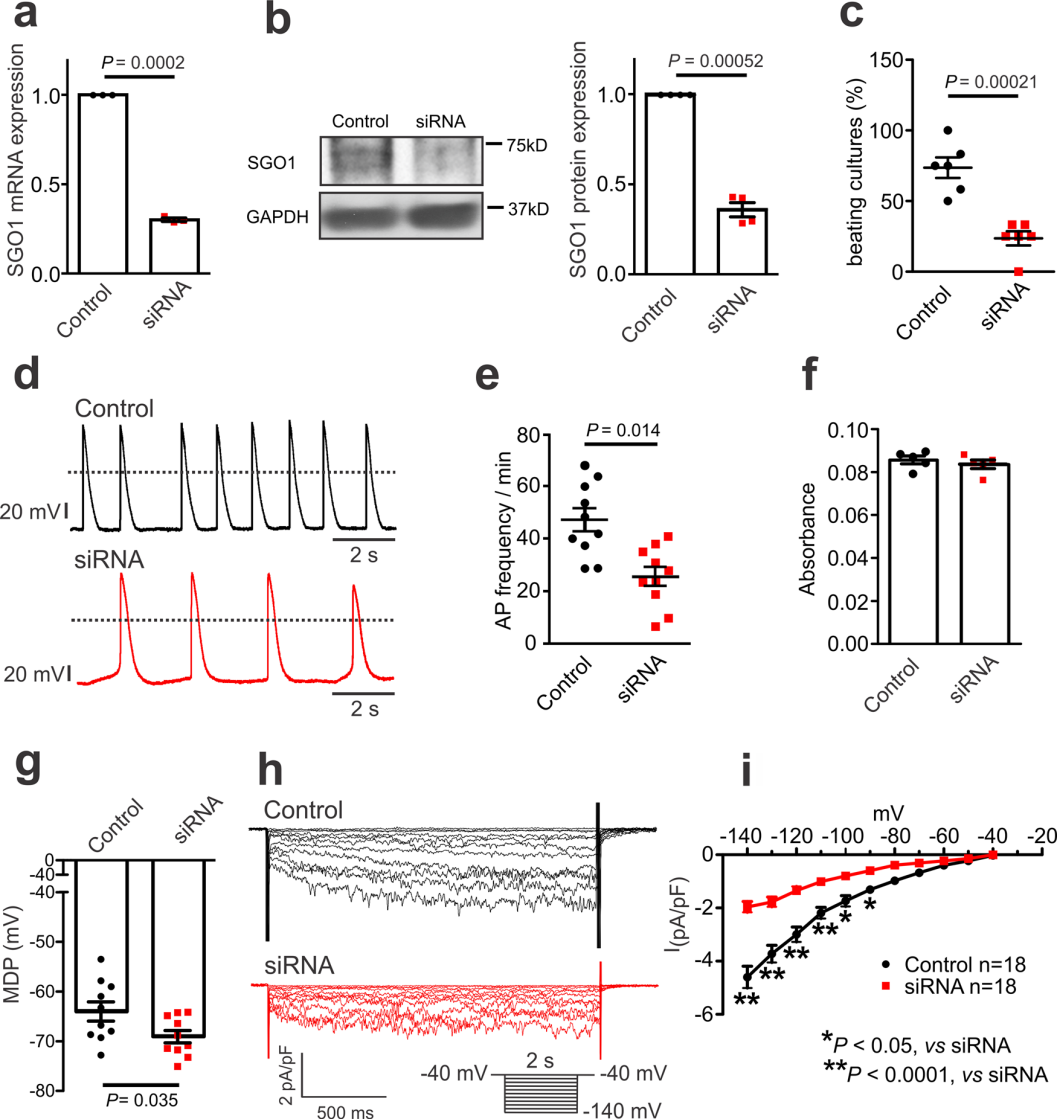
Knockdown of SGO1 suppresses automaticity of NRVMs by inhibiting I_f_. **a** Analysis of SGO1 mRNA expression after 2-day siRNA transduction in NRVMs. *n* = 3 biologically independent preparations exposed to both conditions. Data are expressed as mean ± SEM, normalized to respective control sample result for each preparation. **b** SGO1 protein expression after siRNA transduction in NRVMs. *n* = 4 biologically independent samples. Data are expressed as mean ± SEM. Unpaired two-tailed Student’s *t* test. **c** siRNA targeting SGO1 decreased the number of spontaneously beating NRVM monolayers compared to control after 30 to 48 h of transduction. Each point in the plot represents the percentage of wells of a 24-well plate that were beating for each experiment. *n* = 6 biologically independent experiments. Data are expressed as mean ± SEM. Unpaired two-tailed Student’s *t* test. **d** Representative spontaneous AP recordings in control (black) and siRNA transduced (red) NRVMs. The dashed horizontal line indicates the 0 mV level; the short vertical lines at left are 20 mV scales. **e** siRNA suppressed AP frequency after 2–3 days transduction. *n* = 10 biologically independent samples per group. Data are expressed as mean ± SEM. Unpaired two-tailed Student’s *t* test. **f** Effect of SGO1 knockdown on the viability of NVRMs. Cell viability was assessed with the PrestoBlue assay that measures cellular metabolic reduction. *n* = 5 biologically independent preparations exposed to both conditions. Data are expressed as mean ± SEM, normalized to respective control sample result for each preparation. **g** Effect of SGO1 knockdown on MDP in NRVM monolayers. Data are expressed as mean ± SEM. Unpaired two-tailed Student’s *t* test. **h** Representative I_f_ recordings in control and siRNA transduced NRVMs. **i** I-V relation of I_f_ plotted for control and siRNA transduced NRVMs. Data are mean ± SEM. Two-way ANOVA with Bonferroni’s multiple comparison test. n’s in figure are number of separate cells studied.

Given that SGO1 overexpression promoted NRVM automaticity, we knocked down SGO1 with siRNA to test the role of endogenous SGO1 in NRVMs. SGO1 was knocked down with siRNA transferred via liposomes, with a scrambled siRNA analog used to transfect control NRVMs. SGO1 knockdown by siRNA significantly decreased SGO1 expression at both mRNA and protein levels (Fig. 3a, b). The percentage of beating monolayers was substantially reduced in the siRNA transfected NRVMs compared to scrambled-control NRVMs (Fig. 3c). Next, we recorded the APs in NRVM monolayers of siRNA and control NRVMs (Fig. 3d). SGO1 knockdown significantly suppressed the AP firing frequency (Fig. 3e). In some cancer cell-lines, SGO1-depletion causes G2/M arrest, apoptosis, and mitotic cell death^7,10,16^. As reduced cell number might impair NRVM automaticity, we studied the effect of SGO knockdown on cell viability with the use of PrestoBlue assay. No differences in cell viability were observed between control and siRNA treated NRVMs (Fig. 3f), suggesting that knockdown of SGO1 did not affect NRVM survival under our conditions. The MDP increased from −63.7 ± 2.0 mV in control to −69.0 ± 1.3 mV in siRNA-transfected NRVMs (Fig. 3g), consistent with reduced inward current (like that carried by I_f_). I_f_ was compared at day 2–3 after transfection. SGO1 knockdown significantly suppressed I_f_ current density (Fig. 3h, i), indicating that endogenous SGO1 plays a role in maintaining NRVM automaticity and implicating the pacemaker current I_f_ as playing a critical role in this effect of SGO1.

### SGO1 does not change HCN channel expression in NRVMs

**Fig. 4:**
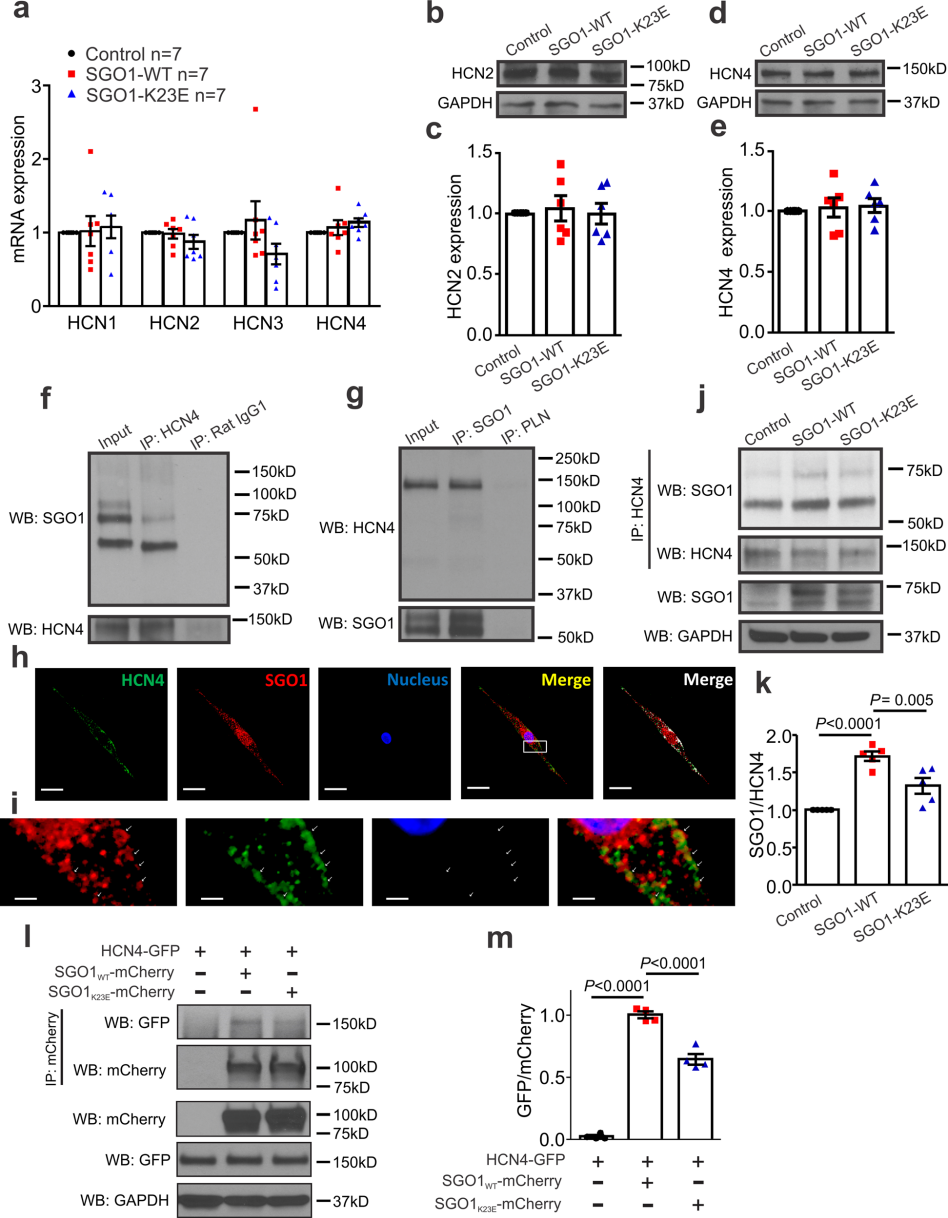
SGO1 interaction with HCN4. **a** SGO1-WT and SGO1-K23E transduction did not change HCN1, HCN2, HCN3, or HCN4 mRNA expression in NRVMs. *n* = 7 biologically independent preparations, each exposed in parallel to each condition. Data are expressed as mean ± SEM, normalized to respective control sample result for each preparation. **b, c** Transduction of SGO1-WT or SGO1-K23E did not affect HCN2 protein expression. *n* = 5 biologically independent preparations, each exposed in parallel to each condition. Data are expressed as mean ± SEM, normalized to respective control sample result for each preparation. **d, e** Transduction of SGO1-WT or SGO1-K23E did not change HCN4 protein expression. *n* = 5 biologically independent preparations, each exposed in parallel to each condition. Data are expressed as mean ± SEM, normalized to respective control sample result for each preparation. **f** Co-IP of native SGO1 with HCN4 in non-transduced NRVMs. NRVM lysates were incubated with HCN4 antibody or rabbit IgG (isotype antibody control); SGO1 antibody was used to detect SGO1 bands. Similar results were obtained in *n* = 5 biologically independent experiments. **g** A reverse co-IP with phospholamban (PLN) antibody as the isotypic antibody control. NRVM lysates were loaded as the input control in **f** and **g**. IP: immunoprecipitation. WB: Western blot. Similar results were obtained in *n* = 5 biologically independent experiments. **h** Confocal images from NRVM slides showing colocalization between native HCN4 and SGO1. Freshly isolated NRVMs were seeded on slides 3 days before photography. The areas of the white zones showing overlapping expression in the merged images are quantified with ImageJ. Scale bar=20 µm. Similar results were obtained in *n* = 3 biologically independent experiments. **i** Magnified view of area indicated in **h**. Scale bar=2 µm. **j** Co-IP to test the interaction with HCN4 in control, SGO1-WT and SGO1-K23E transfected NRVMs. Similar results were obtained in *n* = 5 biologically independent experiments, with each preparation exposed in parallel to each condition. **k** Quantitation of SGO1 and HCN4 interaction in **j**. Data are expressed as mean ± SEM, normalized to respective control sample result for each preparation. One-way ANOVA with Tukey’s multiple comparison test. **l** Loss of interaction between SGO1_K23E_-mCherry and HCN4-GFP in NRVMs. SGO1_WT_-mCherry or SGO1_K23E_-mCherry were co transfected with human HCN4-GFP in NRVMs 2 days before co-IP experiments. mCherry antibody was used to pull down the protein complex and GFP antibody was used to identify HCN4-GFP bands in co-IP. **m** Quantitation of SGO1-HCN4 interaction in (I). *n* = 4 biologically independent experiments, with each preparation exposed in parallel to each condition. Data are expressed as mean ± SEM, normalized to respective control sample result for each preparation. One-way ANOVA with Tukey’s multiple comparison test.

Next, we explored the molecular mechanism underlying SGO1-mediated I_f_ enhancement in NRVMs. The pore-forming subunits responsible for carrying I_f_ are hyperpolarization-activated, cyclic nucleotide-gated cation channel (HCN) channels (HCN1, HCN2, HCN3, and HCN4). HCN4 contributes up to 70–80% of I_f_ in sinoatrial node cells^17–22^. We first considered the possibility that, consistent with the canonical role, SGO1 changes HCN channel expression via direct or indirect epigenetic or transcriptional regulation. However, no difference in HCN1, HCN2, HCN3 or HCN4 mRNA expression was observed in response to SGO1-WT and SGO1-K23E transduction of NRVMs (Fig. 4a). HCN2 and HCN4 show particularly rich protein expression in NRVMs^14^; but neither SGO1-WT nor SGO1-K23E transduction affected HCN2 and HCN4 protein expression (Fig. 4b–e). These results indicate that SGO1 does not alter I_f_ by changing HCN expression.

### SGO1 interacts with HCN4 and K23E impairs the interaction

The mutation (K23E) in CAID-syndrome patients is located in the N-terminal of SGO1 (residues 1-115), which is a highly conserved coiled-coil domain that confers interaction with other cohesin-associated proteins, such as PP2A^5,23^. We therefore considered the possibility that SGO1 can form protein complexes with non-cohesin proteins, such as HCN4, to affect I_f_. We conducted co-immunoprecipitation (co-IP) experiments to assess potential interactions between SGO1 and HCN4 in non-transfected native NRVMs. The SGO1-HCN4 complex was pulled down by a rabbit polyclonal antibody against HCN4 and SGO1 bands were detected with a mouse monoclonal antibody against SGO1, which we previously validated to specifically label SGO1 in human and mouse^5,24^ (Fig. 4f). Two bands were found between 75 kD and 50 kD, corresponding to SGO1 immunoblots in previous reports^25,26^. These bands were suppressed with siRNA in SGO1 knockdown experiments, confirming their identity (Fig. 3b). The corresponding bands were absent in isotype control IgG antibody samples (Fig. 4f). We also isolated the SGO1-HCN4 complex with the antibody directed against SGO1, observing HCN4 bands at the expected position of ~130 kD (Fig. 4g). The SGO1 band was absent in the isotype antibody control (mouse monoclonal antibody against phospholamban (PLN)) lane (Fig. 4g). These results indicate that SGO1 interacts with HCN4 in NRVMs. Consistent with this notion, immunofluorescence (IF) staining revealed that while SGO1 was strongly concentrated in the nucleus, SGO1 also colocalized with HCN4 at the cell membrane of NRVMs (Fig. 4h). The white area in the upper panel indicates the overlapping area merged by the Manders overlap method calculated through ImageJ Software; the Manders Overlap Coefficient (MOC) was 71.4 ± 5.3 (*n* = 5). High magnification showed the overlapping staining for SGO1 and HCN4 (Fig. 4i). No staining was observed in the negative control obtained by omitting primary antibody (Supplementary Fig. 1). Together, the functional changes in pacemaker currents, along with molecular interaction revealed by co-IP and supported by IF, support a functional non-canonical physical interaction between SGO1 and HCN4.

As overexpression of human SGO1-WT enhanced NRVM automaticity (Fig. 1), we tested its interaction with native HCN4 under similar conditions. Co-IP showed that transduction with human SGO1-WT increased the amount of SGO1 interacting with rat HCN4 (Fig. 4j, k). SGO1 enhances NRVM automaticity by interacting with rat HCN4. To further evaluate the interaction between SGO1 and HCN4, we labeled human HCN4 with green fluorescent protein (GFP) and labeled human SGO1 with mCherry, then assessed the interaction between the two fusion proteins (HCN4-GFP and SGO1_WT_-mCherry) in NRVMs. Rat monoclonal antibodies against mCherry were used to pull down the protein complex and mouse monoclonal antibodies against GFP were used for immunodetection. Immunoprecipitation with mCherry antibodies revealed co-IP of HCN4-GFP and SGO1_WT_-mCherry in co-expressing NRVMs (Fig. 4l, m). The mutant (SGO1-K23E) showed reduced binding with HCN4 in both the native HCN4-SGO1 co-IP test system (Fig. 4j–k) and the fusion protein co-IP system (Fig. 4l–m), indicating that the mutation (K23E) causes reduced interaction with HCN4.

### SGO1 enhances HCN4 cell-surface expression

**Fig. 5:**
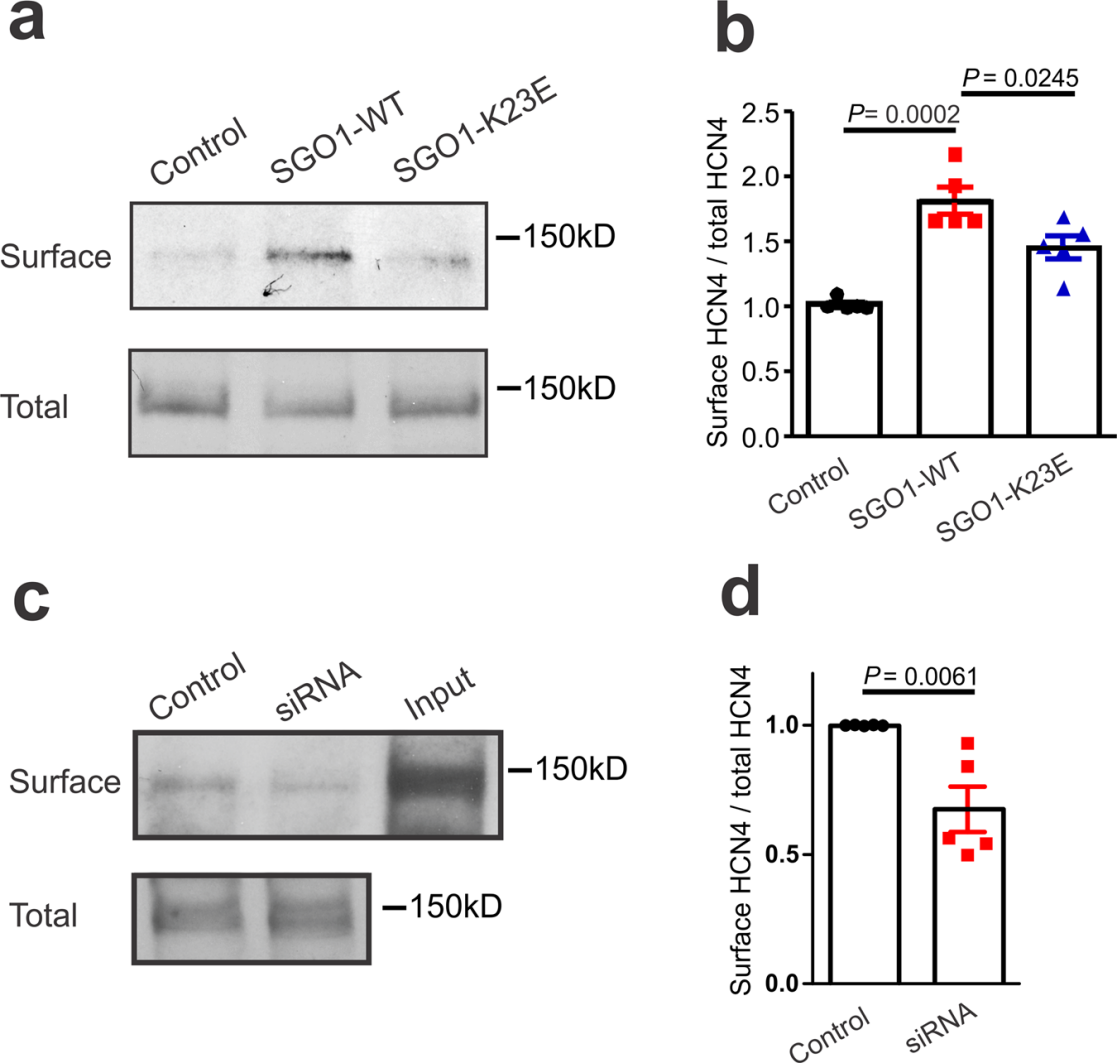
Effect of SGO1-WT and SGO1-K23E on HCN4 surface expression in NRVMs. **a** SGO1-WT and SGO1-K23E were transduced into NRVMs 2 days before surface labeling with biotin probe. The surface and total expression of HCN4 was tested by Western blot. **b** Quantitation of HCN4 surface expression in **a**. *n* = 5 biologically independent preparations exposed to all conditions. Data are expressed as mean ± SEM, normalized to respective control sample result for each preparation. One-way ANOVA with Tukey’s multiple comparison test. **c** The surface and total expression level of HCN4 were tested after 2 days of siRNA and control scrambled RNA transduction. NRVMs lysates were loaded as input control. **d** Quantitation of HCN4 surface expression in **c**. *n* = 5 biologically independent preparations exposed to both conditions. Data are mean ± SEM, normalized to respective control sample result for each preparation. Unpaired two-tailed Student’s *t* test.

We then addressed HCN4 localization at the cell surface in NRVMs with the use of a biotinylation assay system. Although total HCN4 expression was not affected by SGO1 transduction (Fig. 4d, e), SGO1 transduction increased HCN4 expression on the cell-surface (Fig. 5a, b). Consistent with its reduced interaction with HCN4, SGO1-K23E showed reduced ability to enhance HCN4 surface localization in NRVMs (Fig. 5a, b). Knockdown of SGO1 in NRVMS also reduced HCN4 cell surface expression (Fig. 5c, d).

### HCN4 does not interact with PP2A in NRVMs

SGO1 canonically enhances cohesin stability by recruiting the phosphatase PP2A and preventing phosphorylation-mediated opening of the cohesin complex^27^. Since HCN4 function is also modulated by phosphorylation^28^, we asked whether SGO1 and HCN4 form a macromolecular complex with PP2A. However, while co-IP for PP2A and SGO1 showed the previously reported strong interaction, co-IP for PP2A and HCN4 did not show co-precipitation (Supplementary Fig. 2), indicating that SGO1 regulation of HCN4 function is not related to a direct interaction with PP2A.

### CAID-patient hiPSC-CMs exhibit dysrhythmic activity

**Fig. 6:**
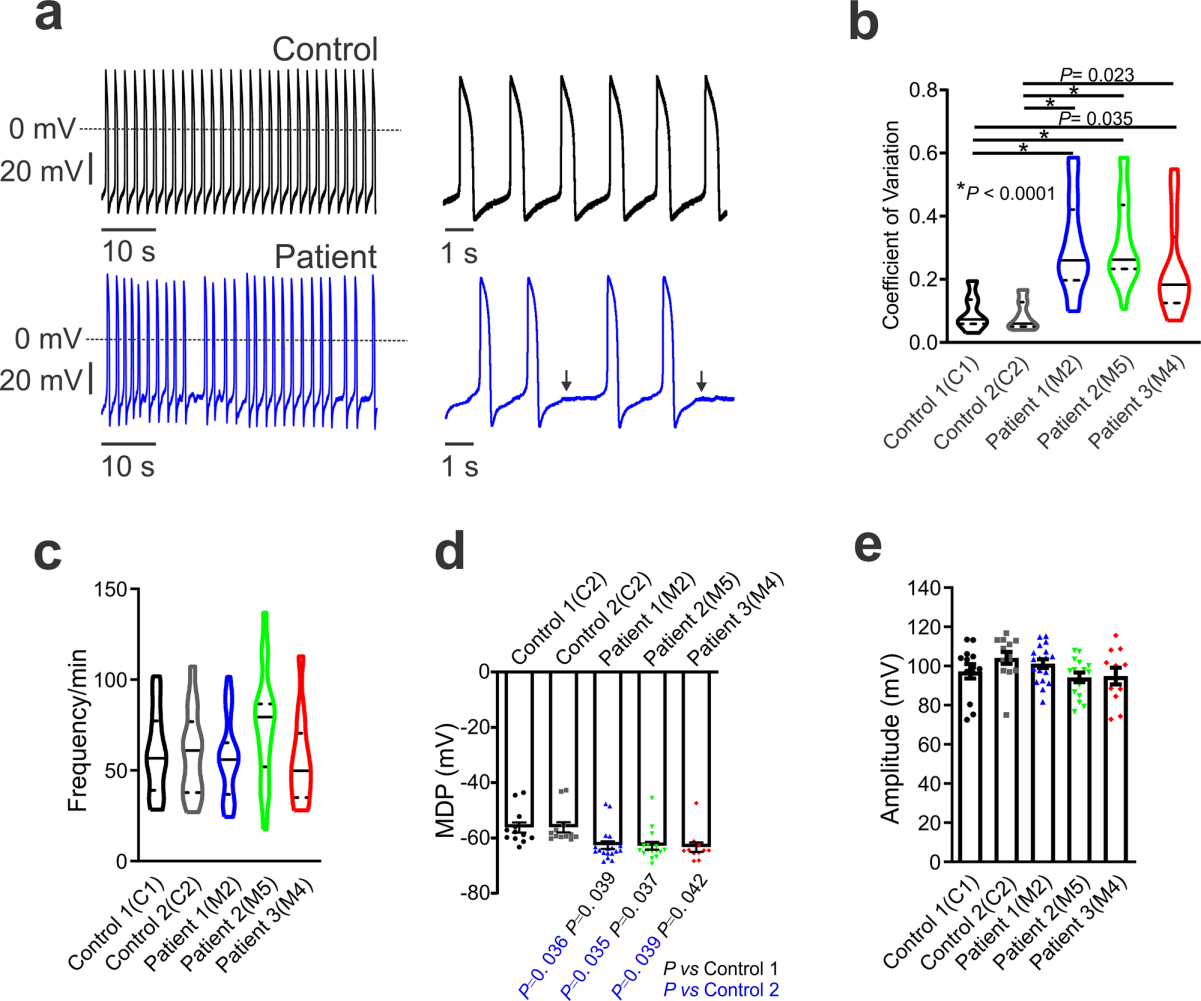
Pacemaker stability is disrupted in CAID-patient hiPSC-CMs. **a** Spontaneous action-potential recordings from control (C1, black) and patient (M2, blue) single hiPSC-CMs. The short vertical lines at left are 20 mV scales. **b** Analysis of coefficient of variation of action potential firing in healthy control and CAID patient-specific hiPSC-CMs. *n*_C1_ = 14, *n*_C2_ = 13, *n*_M2_ = 24, *n*_M5_ = 19, and *n*_*M4*_ = 12 biologically independent cells. Data are represented as violin plots, with median (solid line) and interquartile range (dotted line). One-way ANOVA with Tukey’s multiple comparison test. **c** Spontaneous action potential frequency in control and CAID-patient hiPSC-CMs. *n*_C1_ = 14, *n*_C2_ = 13, *n*_M2_ = 24, *n*_M5_ = 19, and *n*_*M4*_ = 12 biologically independent cells. Data are represented as violin plots, with median (solid line) and interquartile range (dotted line). There was one cell without meaningful action potentials in control 1 (C1), one cell in patient 1 (M2) and 1 cell in patient 2 (M5); these were not analyzable for frequency and were not included in analyses. **d** MDP in control and CAID-patient hiPSC-CMs. *n*_C1_ = 12, *n*_C2_ = 12, *n*_M2_ = 18, *n*_M5_ = 16, and *n*_*M4*_ = 11 biologically independent cells. Data are expressed as mean ± SEM. One-way ANOVA with Tukey’s multiple comparison test. **e** Action potential amplitude in control and patient hiPSC-CMs. *n*_C1_ = 12, *n*_C2_ = 15, *n*_M2_ = 18, *n*_M5_ = 16, and *n*_*M4*_ = 11 biologically independent cells. Data are expressed as mean ± SEM.

The availability of patient-derived human induced pluripotent stem-cell derived cardiomyocytes (hiPSC-CMs) has allowed for powerful in vitro exploration of disease-specific pathophysiological mechanisms^29–33^. We therefore tested whether the effects of the SGO1-K23E mutation observed in the NRVM system are also present in CAID patient-derived hiPSC-CMs. A total of 5 lines of hiPSCs were generated from 2 unrelated healthy controls (C1 and C2) and 3 CAID syndrome patients (M2, M4, and M5) harboring the homozygous c.69 A > G variant in *SGO1* (Supplementary Fig. 3a). Pluripotency of the generated hiPSC lines were comparable, as assessed by the mRNA levels of pluripotency markers like OCT3/4, SOX2, and NANOG (Supplementary Fig. 3b). First, we studied the spontaneous rhythm of hiPSC-CMs. Spontaneous APs were recorded in single hiPSC-CMs (Fig. 6a). We found that the CAID patient hiPSC-CMs (M2, M4, and M5) exhibited dysrhythmic activity in the form of increased variability in the interbeat interval (Fig. 6a). The coefficient of variation of peak-peak spontaneous AP intervals of CAID-patient hiPSC-CMs was significantly increased compared to those of the healthy controls (C1 and C2) (Fig. 6b), recapitulating dysrhythmic phenotypes seen in CAID-syndrome. This kind of dysrhythmic activity was also observed in atrial-like spontaneous APs in CAID-patient hiPSC-CMs (Supplementary Fig. 4a, b). Irregular and/or slow rhythms are typical of sinoatrial node dysfunction observed in families and mice with sinus node disease due to HCN mutations, as well as in mice with sinoatrial node dysfunction due to NCX1 knockout^18–22,34,35^. To relate our in vitro observations to in vivo findings, we reviewed ECG data from CAID-patients. We were able to obtain baseline 12-lead ECGs from 2 CAID-patients with marked sinus rhythm arrhythmia, one of whom donated the hiPSC-CM line M4 used in this study (Supplementary Fig. 5). Ultrashort heart rate variability analysis^36^ showed largely increased heart rate variability, with root mean square of successive differences (RMSSD) values of 327 and 295 ms respectively, versus normal values of the order of 28–29 ms^36^.

Membrane oscillations resembling delayed afterdepolarization (DADs) were observed during longer diastolic intervals for CAID-patient hiPSC-CMs (Fig. 6a and Supplementary Fig. 4a, arrows). These afterdepolarizations appeared never to reach threshold to cause arrhythmic activity. Despite the unstable dysrhythmic activity in CAID-patient hiPSC-CMs, slower rates were not noted in CAID-patient hiPSC-CMs (Fig. 6c). The MDP in CAID-patient hiPSC-CMs shifted to more negative potentials compared with healthy controls (Fig. 6d). No differences were noted in AP amplitude (Fig. 6e) between controls and CAID-patient hiPSC-CMs.

### CAID-patient hiPSC-CMs exhibit decreased I_f_

**Fig. 7:**
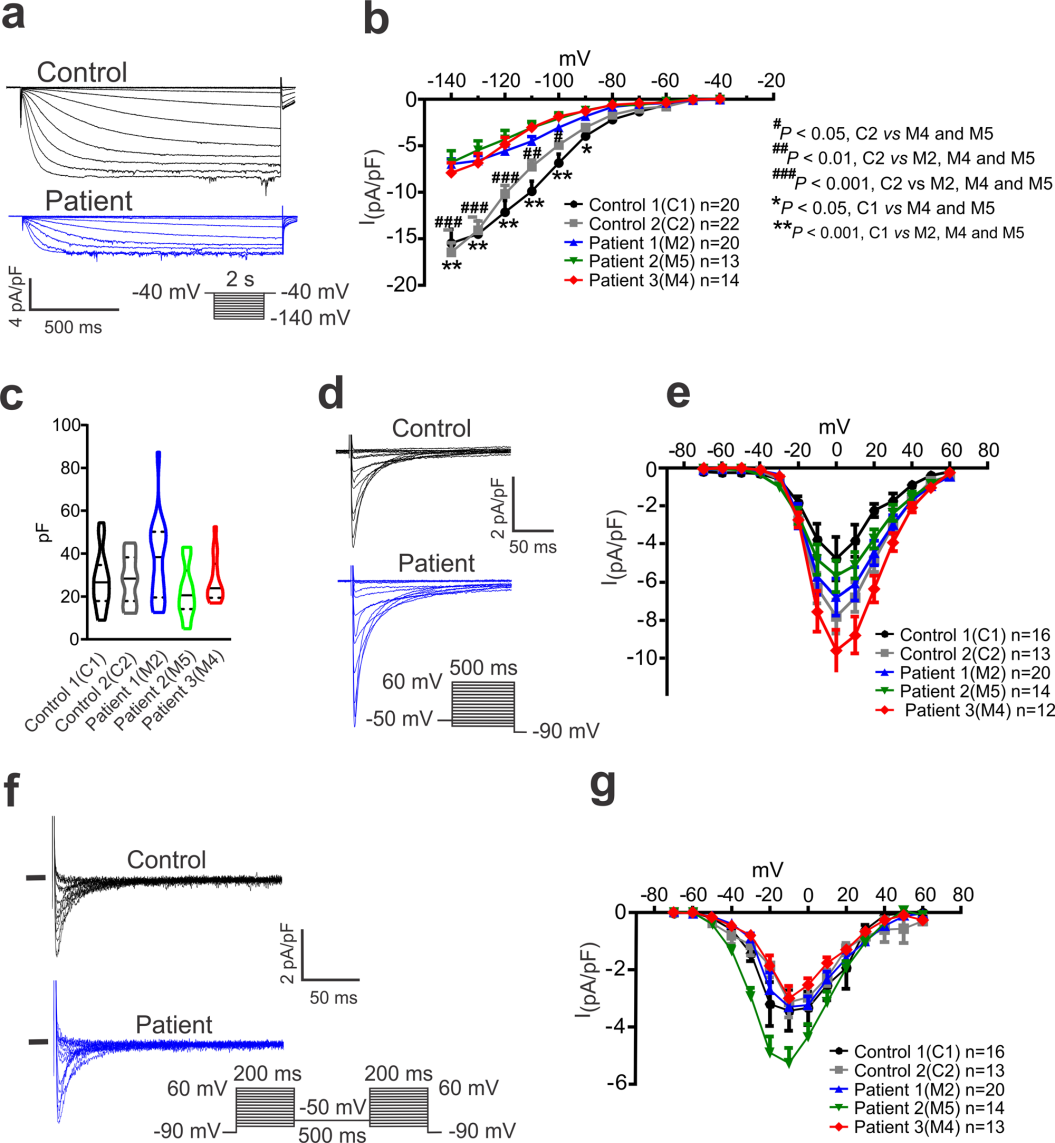
I_f_ is smaller in CAID-patient hiPSC-CMs compared to controls. **a** Representative I_f_ recordings from control (C1) and CAID patient (M2) hiPSC-CMs. **b** I-V relation of I_f_ in hiPSC-CMs. Data are mean ± SEM. Two-way ANOVA with Bonferroni’s multiple comparison test. Cell capacitance of hiPSC-CMs for I_f_ recordings shown in **b**. Data are represented as violin plots, with median (solid line) and interquartile range (dotted line). **c** Capacitance data for cells used to analyze I_f_ (violin plots). **d** I_CaL_ recordings from control (C1) and patient (M2) hiPSC-CMs. **e** I-V relations of I_CaL_ recorded in control and CAID-patient hiPSC-CMs. Data are mean ± SEM. **f** I_CaT_ recordings in control (C1) and CAID-patient (M2) hiPSC-CMs. **g** I-V relations of I_CaT_ recorded in control and CAID-patient hiPSC-CMs. Data are mean ± SEM.

We also compared I_f_ between control and CAID-patient hiPSC-CMs. The hiPSC-CMs differentiated with our protocol showed robust I_f_ (Fig. 7a). The I_f_ density in CAID-patient hiPSC-CMs (M2, M4 and M5) was decreased compared to the healthy control hiPSC-CMs (C1 and C2) (Fig. 7b). No differences were observed in cell-capacitance between the control and patient hiPSC-CMs (Fig. 7c). These results are consistent with the idea that the SGO1-K23E mutation causes pacemaker dysfunction via reduced I_f_ in CAID patients.

We also tested the effects of the K23E mutation on I_CaL_ and I_CaT_ in CAID-patient hiPSC-CMs. Typical I_CaL_ recordings were obtained in control and CAID-patient hiPSC-CMs (Fig. 7d). There were no statistically significant changes in I_CaL_ current-voltage relations (Fig. 7e). Typical I_CaT_ was also observed in hiPSC-CMs (Fig. 7f). For only one patient (M5) did hiPSC-CMs showed a larger current density than control or other CAID-patient hiPSC-CMs (Fig. 7g). Taken together, these data suggest that the mutation do not affect automatic activity in CAID-patient hiPSC-CMs via changes in I_CaL_ or I_CaT_.

### CAID-patient hiPSC-CMs show reduced interaction with HCN4

**Fig. 8:**
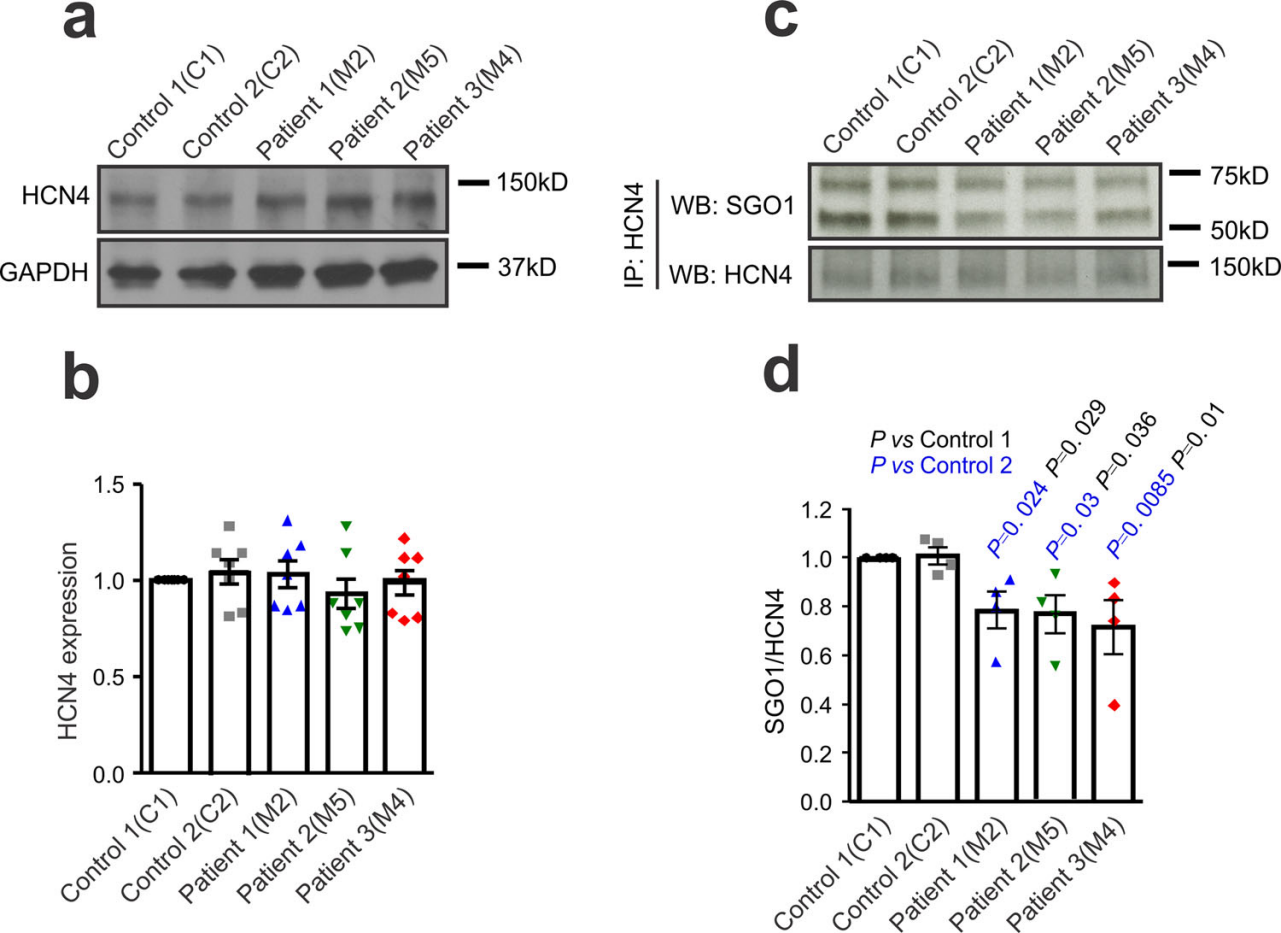
CAID-patient hiPSC-CMs show a suppressed interaction between SGO1 and HCN4. **a** Western blots for HCN4 protein expression in healthy control and CAID-patient hiPSC-CMs. **b** Western blot analysis of HCN4 protein expression. *n* = 7 independent experiments with cells from each line. Data are expressed as mean ± SEM. **c** Comparison of the interaction between SGO1 and HCN4 in healthy control and CAID-patient hiPSC-CMs. **d** Analysis of interaction between SGO1 and HCN4 data of the type shown in **c**. *n* = 4 independent experiments with cells from each line. Data are expressed as mean ± SEM. One-way ANOVA with Tukey’s multiple comparison test.

In NRVMs, we found that SGO1-K23E partially lost the physical interaction with HCN4. To confirm this result in human cardiomyocytes, we studied HCN4 expression and the SGO1-HCN4 interaction in hiPSC-CMs. Consistent with the results in NRVMs, we did not find any difference in total HCN4 expression between control and CAID-patient hiPSC-CMs (Fig. 8a, b). In co-IP experiments, CAID-patient hiPSC-CMs (M2, M5, M4) showed lesser interaction with HCN4 than observed in control lines (C1, C2) (Fig. 8c, d), further supporting the idea that the mutant (SGO1-K23E) partly loses the interaction with HCN4. These results are consistent with the idea that patients with the K23E mutation show SA node dysfunction because of impaired SGO1 interaction with HCN4.

## Discussion

In this study, we have identified a mechanism controlling cardiac automaticity. The pacemaker current I_f_ is a crucial determinant of the diastolic depolarization leading to spontaneous cardiac rhythmic activity, including that in the SA node pacemaker^17,37^. We show here that overexpression of exogenous SGO1 enhances I_f_ and automaticity of NRVMs, while knockdown of endogenous SGO1 suppresses I_f_ and automaticity. Furthermore, evidence of physical association between SGO1 and HCN4 is observed in NRVMs for the native proteins, as well as with exogenous fusion proteins SGO1-GFP and HCN4-mCherry, indicating a physical interaction between SGO1 and HCN4. Although total HCN4 expression is not affected by SGO1 transduction, SGO1 transduction increases HCN4 cell-surface expression in NRVMs. Knockdown of endogenous SGO1 also decreases HCN4 cell surface expression. The CAID mutant SGO1-K23E has reduced ability to support the physical interaction with HCN4 and to regulate I_f_. Taken together, the NRVM data suggest that this non-canonical, cohesin-independent SGO1-HCN4 interaction regulates HCN4 surface expression and thereby cardiomyocyte automaticity.

In order to confirm the unique cytosolic, non-canonical role for SGO1 in a human system, we used a disease-specific hiPSC-CM model for this inherited human cardiac pacemaker disease. Human iPSC-CMs maintain pacemaker activity and underlying mechanisms, which we exploited to recapitulate and study the consequences of the CAID Syndrome on human cardiac pacemaking in vitro. Our results confirmed that the non-canonical interaction between HCN4 and SGO1 is likely responsible for pacemaker dysfunction in CAID Syndrome. A variety of genetic cardiomyopathies have been studied before in patient-derived hiPSC-CMs^38^, but our study is to our knowledge the first to analyze the mechanism of a genetic cardiomyopathy manifesting primarily as pacemaker dysfunction in this system.

I_f_ function is subject to substantial biochemical regulation. In addition to the well-recognized role of cAMP-activated protein-kinase A, Src-kinase mediated tyrosine-phosphorylation, direct cAMP-binding, control by phosphoinositides including phosphatidylinositol-4,5-bisphosphate, a whole range of phosphatases and AMPK signaling are all potentially important modulators of HCN-channel function in mammalian hearts^28,39–42^. Although we found that HCN4 does not form complexes with PP2A in NRVMs, implying that PP2A-related dephosphorylation of HCN channels may not participate in SGO1 mediated HCN4 regulation, a whole range of other signaling pathways should be assessed for their potential involvement in I_f_-regulation in the CAID Syndrome. In addition to direct regulation of HCN-function, such modulation can also occur by altering the function of chaperone-proteins or other factors controlling HCN-subunit trafficking to the membrane.

We also found that both SGO1-WT and SGO1-K23E enhance I_CaT_, which contributes to late diastolic depolarization^15^, in NRVMs. The mechanisms of this action are not clear, but given the similar effects of WT and mutant proteins, it is likely different from the mechanisms involved in I_f_-upregulation. Taken together with the unchanged I_CaT_ in CAID-patient hiPSC-CMs, these observations suggest that I_CaT_ is an unlikely mediator for SGO1 effects on cardiac automaticity in CAID.

Here, we showed that SGO1 is a key regulator of cardiac pacemaker function. However, the CAID Syndrome involves both cardiac pacemaking dysfunction and intestinal pseudo-obstruction. Interstitial Cells of Cajal, which function as pacemaker cells in the gut, also express HCN4, which has been reported to be a selective marker for this cell-type in mice^43^. There is evidence that intact HCN2 is required for normal intestinal peristalsis in a genetically-defined mouse model and that HCN4 is required for retrograde peristalsis in zebrafish^44,45^. Further work is needed to clarify whether SGO1 interacts with intestinal HCN channels and, if so, whether disruption of this interaction might contribute to the intestinal pseudo-obstruction component of CAID syndrome.

Both the “membrane clock”, in which HCN channels play a central role, and the “calcium clock” (involving sarcoplasmic-reticulum (SR) Ca^2+^-release, the sodium-calcium exchanger (NCX) and SR Ca^2+^-uptake) take part in cardiac pacemaking^14,15,46–49^. In this study, we focused on the channels involved in the “membrane clock” mechanism because of clear changes in I_f_. However, we cannot exclude effects mediated by changes in the calcium clock. Further work is needed to evaluate the potential involvement of other factors that might affect pacemaking in CAID patients.

In summary, we report a non-canonical role for SGO1, through which SGO1 and SGO1-K23E appear to affect I_f_ in a direct physical, and cohesin-independent fashion. SGO1 directly interacts with HCN4 and enhances HCN4 cell surface expression, thereby regulating I_f_ and pacemaker activity. The K23E mutation in SGO1 results in a loss of interaction between SGO1 and HCN4, impairs HCN4 surface expression, reduces I_f_ and disturbs pacemaker activity. These findings shed light on the basis for cardiac pacemaker dysfunction in CAID Syndrome and suggest that improving HCN4 cell surface expression via SGO1 could be a therapeutic approach for pacemaking disorders.

## Methods

### NRVM isolation and culture

All animal-handling procedures followed the National Institutes of Health guidelines and were approved by the Montreal Heart Institute Animals Research Ethics Committee. Neonatal rat ventricular myocytes were obtained from 1 or 2 day old Wistar rat pups (Charles River Laboratories). Neonatal rat pup sex was not determined. Hypothermia was utilized to anesthetize rat pups before they were euthanized by decapitation. The Neonatal Cardiomyocyte Isolation System (Worthington Biochemical) was utilized to obtain single cardiomyocytes. To form a monolayer, the NRVMs were plated at a density of 20,000 cells per cm^2^ surface area in 24-well plates or 4 mm×10 mm coverslips. In the first 6 h, the cells were cultured in a routine cardiomyocyte medium based on M199 containing 10% FBS and 100 U/ml penicillin/streptomycin, then used for lentivirus transduction in medium with 5% FBS. After 24-h transduction, we refreshed the medium with medium containing 10% FBS and penicillin/streptomycin. After 48-h culture, action potentials were recorded from the NRVM monolayer cultures on coverslips. Single cells were digested from the monolayers with trypsin-EDTA and used for I_f_ and I_Ca_ recording.

### Lentivirus construction and purification

The cDNAs of human *HCN4*, human wild type (SGO1-WT, NM_001199252.3) and mutant (SGO1-K23E) *SGO1* were subcloned into the lentiviral vector pLentiCMV in which the GFP coding section was deleted from the backbone of pLentiCMV-GFP-Neo (Addgene #17447, gift from Eric Campeau & Paul Kaufman). The GFP was cloned into the N-terminal of HCN4 and mCherry was cloned into the N-terminal of SGO1-WT and SGO1-K23E to form fusion proteins. The lentiviral plasmids carrying SGO1-WT, SGO1-K23E, HCN4, SGO1-WT-mCherry, SGO-K23E-mCherry, or HCN4-GFP were co-transfected into HEK293T cells with packaging plasmids pMD2.G (Addgene #12259) and psPAX2 (Addgene #12260), which were gifts from Didier Trono. The supernatants containing virus were harvested 48 and 72 h after transfection and concentrated using Lenti-X^TM^ concentrator (TaKaRa Bio). The viral pellet was resuspended in serum-free DMEM medium and viral titers were measured with QuickTiter Lentivirus Quantitation Kit (Cell Biolabs). Viral stocks were stored at –80 °C until use.

### SGO1 overexpression and knockdown on NRVMs

Lentiviruses were used to overexpress wild type SGO1 and mutant SGO1 (K23E) in NRVMs. The cells were cultured for 6 h to permit cell attachment to the coverslips or plates. For overexpression experiments, the lentiviruses were added to NRVM cultures and incubated for 24 h. The medium was then refreshed with normal medium lacking lentivirus. MOI = 50 was used in overexpression experiments. To knock down SGO1 in NRVMs, the medium was refreshed into Opti-medium without FBS before transfection. The cells were then transfected with siRNA or scrambled RNA at 100 nM per well in 24-well plates with Lipofectamine RNAiMAX (Life Technologies). After 24 h, the Opti-medium was replaced by M199 with 10% FBS. The siRNA (Stealth RNAi™ siRNA Duplex, Invitrogen) sequences are listed below (5ʹ−3ʹ): 1. UUUCAGUGUACACAGCUGGCAGGUG, 2. CACCUGCCAGCUGUGUACACUGAAA. Stealth RNAi™ siRNA Negative Control Med GC Duplex (Invitrogen, #12935112) was used as scrambled control RNA.

### Electrophysiology

Whole cell patch-clamp recordings were performed with an Axopatch 200B amplifier (Axon instruments, USA). Data were sampled at 10 Hz and filtered at 5 kHz. All recordings were obtained at 36 °C. Cell capacitance was calculated from the time constant of a capacitative current elicited by a 5 mV depolarization from −60 mV. Borosilicate glass microelectrode tip resistances were 5–8 MΩ. Action potential (AP) recordings were obtained with nystatin perforated patch-clamp pipettes with I = 0 mode as described previously^50^. The cells were superfused with Tyrode Solution containing NaCl 136 mM, KCl 5.4 mM, NaH_2_PO_4_ 0.33 mM, CaCl_2_ 1.8 mM, MgCl_2_ 1 mM, glucose 10 mM, and Hepes 5 mM (pH 7.4, NaOH). The internal solution contained KCl 120 mM, MgCl_2_ 1 mM, Mg-ATP 3 mM, EGTA 10 mM, Hepes 10 mM (pH7.2, KOH). For I_f_ recording, the standard whole cell tight seal patch-clamp method was used. To block I_K1_, I_CaL_, I_CaT_ and I_to_, 2 mM BaCl_2_, 0.2 mM CdCl_2_, 2 mM NiCl_2_ and 4 mM 4-aminopyridine (4-AP) were added to Tyrode solution and with the same internal solution as used for AP recording^51^. The external solution for I_Ca_ recording contained tetraethylammonium chloride (TEA-Cl) 136 mM, CsCl 5.4 mM, CaCl_2_ 2 mM, MgCl_2_ 0.8 mM, Hepes 10 mM, glucose 10 mM and 4-AP 2 mM (pH 7.4, CsOH). The internal solution for I_Ca_ recording contained CsCl 120 mM, TEA-Cl 20 mM, MgCl_2_ 1 mM, Mg-ATP 5 mM, Li-GTP 0.1 mM, EGTA 10 and Hepes 10 (pH7.3, CsOH)^52^. I_CaL_ and I_CaT_ were separated with the use of selective voltage protocols as shown in Fig. 2. All electrophysiological data were collected with Clampex 10.4.

### Western blot

Western blot analysis was performed on Mini-PROTEAN^®^ TGX™ Gels (Bio-Rad Laboratories). Proteins were electrotransferred onto Immobilon-P PVDF membrane (millipore, pore size 0.45 mm). After blocking 1 h in Tris-HCl-buffered saline with 0.2% Tween-20 (TBST) and 5% non-fat milk (Bioshop^®^ Canada), the membranes were incubated overnight with primary antibody in TBST containing 2% non-fat milk. The rabbit polyclonal anti-HCN2 antibody (1:500; APC-030, Alomone Labs), rabbit polyclonal anti-HCN4 antibody (1:1000; APC-052, Alomone Labs), mouse monoclonal anti-SGO1 antibody (1:1000; ab58023, Abcam), mouse monoclonal anti-GAPDH antibody (1:1000; 10R-G109a, Fitzgerald), mouse monoclonal anti-GFP antibody (1:1000; MA5-15256, Invitrogen), rat monoclonal anti-mCherry (1:1000; M11217, Invitrogen) and mouse monoclonal anti-PP2A C subunit (1:1000; SAB4200266, Sigma-Aldrich) were utilized to detect HCN2, HCN4, SGO1, GAPDH, GFP, mCherry and PP2A C proteins respectively. Detection was carried out by the use of horseradish peroxidase conjugated secondary antibodies (Donkey anti rabbit secondary antibody, 1:5000, #711035152, Jackson; Donkey anti mouse secondary antibody, 1:5000, #711035151, Jackson; Donkey anti rat secondary antibody, 1:5000, #A18745, Invitrogen), photographic film (Agfa NV) and the Western Lighting^®^ plus ECL kit (PerkinElmer).

### RNA extraction and TaqMan real-time quantitative polymerase chain reaction (qPCR)

RNA was isolated with NucleoSpin^®^ RNA Kits (Macherey-Nagel). First-strand complementary DNA was synthesized from 2 μg of total RNA with high-capacity cDNA Reverse Transcription Kit (Applied Biosystems). Real-time qPCR was carried out with carboxy-fluorescein (FAM)-labeled fluorogenic TaqMan assay primers and TaqMan Universal Master Mix (Applied Biosystems). Fluorescence signals were detected with StepOnePlus™ Real-Time PCR System (Applied Biosystems) in duplicate and relative quantities (2^-ΔCt^) were calculated with the geometric mean of the reference gene GAPDH as an internal standard. TaqMan^®^ Assay primers (ThermoFisher) used for testing HCN1, HCN2, HCN3, HCN4 and GAPDH are list in Supplementary Table 1.

### Immunofluorescence (IF)

Cells were plated on 0.2% gelatin-coated coverslips and cultured for 3 days. Cultured cells were washed twice with 0.1 M phosphate-buffered saline (PBS) and fixed with paraformaldehyde (4%) in PBS for 10 min at room temperature, followed by several washes in PBS. Cells were subsequently incubated in PBS containing 2% normal goat serum for 30 min to block non-specific binding. They were then incubated with rabbit polyclonal anti-HCN4 antibody (1:200; APC-052, Alomone Labs) mixed with mouse monoclonal anti-SGO1 (1:200; ab58023, Abcam) in 2% normal goat serum PBS at 4 °C overnight. After washing with PBS, the cells were incubated with Alexa 488-conjugated goat anti-rabbit secondary antibody (1:1000; A-11078, Thermo Fisher Scientific) and an Alexa 555-conjugated goat anti-mouse secondary antibody (1:1000; A-21422, Thermo Fisher Scientific) for 1 h at room temperature. Nuclei were stained with 1 µM TOPRO3. Negative controls were performed by omitting the primary antibodies from the incubation solution. The fluorescence images were obtained with a Zeiss LSM‐710 inverted confocal laser scanning microscope (Carl Zeiss Meditec GmbH, Germany) and ZEN2.6 software was utilized for image collection.

### Co-Immunoprecipitation (co-IP)

NRVMs or hiPSC-CMs were harvested in ice-cold co-IP buffer containing 50 mM Hepes buffer (pH 7.4), 10% glycerol, 150 mM NaCl, 1% Triton X-100, 0.5% Nonidet P-40, 1 mM EDTA, 1 mM EGTA, protease inhibitor cocktail (B14012, Bimake), 1 mM phenylmethylsulfonyl fluoride, and 1 mM Na_3_VO_4_. The cells were treated homogenized with an ultrasonic device. The homogenates were centrifuged at 12,000 g for 20 min at 4 °C. Supernatants were collected and protein concentration was determined by the Bio-Rad Protein Assay Dye Kit (#5000006, Bio-Rad Laboratories). Cell homogenates (400 μg of protein) were preincubated for 1 h with 20 μl of protein G-Sepharose CL-4B (Amersham Biosciences) at 4 °C and centrifuged to remove proteins that had adhered nonspecifically to protein G. In total, 4 μg primary antibodies bound to and then were cross-linked to Dynabeads Protein G (10003D, Thermo Fisher Scientific) with 20 mM dimethyl pimelimidate (DMP). The supernatants were incubated with the Dynabeads-protein G-antibody complex overnight at 4 °C. The supernatant was removed by magnet and the pellets were washed three times with co-IP buffer without glycerol. Bound proteins were eluted by boiling at 60 °C for 9 min in 2×SDS-PAGE loading buffer and then isolated by magnet. The proteins in supernatants were assayed by Western blot. Rabbit polyclonal anti-HCN4 antibody (APC-052, Alomone Labs) and mouse monoclonal anti-SGO1 antibody (ab58023, Abcam) were utilized to pull down the protein complex. As an isotype control, mouse monoclonal anti-phospholaman (PLN) antibody (MA3-922, Thermo Fisher Scientific), mouse monoclonal anti glycogen synthase 1 antibody (sc-81173, Santa Cruz Biotechnology) and normal rabbit IgG (#12370, Sigma-Aldrich) were used in co-IP experiments.

### Cell surface expression

NRVMs were washed with Dulbecco’s Phosphate-Buffered Saline (DPBS) containing Mg^2+^ and Ca^2+^ at pH 7.3. Membrane proteins were biotinylated by incubating cells with 2 mg/mL of EZ-link^TM^ sulfo-NHS-LCLC-biotin (Thermo Fisher Scientific) in DPBS for 30 minutes at 4 °C with occasional shaking. The cells were then rapidly washed with DPBS 3 times, then washed with DPBS containing 50 mM glycine to allow neutralization of non-crosslinked biotin reagents. The cells were then scraped into IP buffer and rotated for 1 h at 4 °C. The insoluble materials were removed by centrifugation (14,000 × *g* × 20 min). The supernatants were mixed with Ultralink Immobilised NeutrAvidin beads (Thermo Fisher Scientific) and rotated overnight at 4 °C. The co-IP buffer without glycerol was used to wash the beads for 45 min. Precipitated beads were resuspended in 2×SDS-PAGE loading buffer and heated for 9 min at 60 °C. Subsequently, Western blots were performed to analyze biotinylated HCN4.

### Generation and Maintenance of hiPSCs

Human induced pluripotent stem cells (hiPSC) were derived from two healthy donors: C1 (SJi3252C2) and C2 (SJ3013C2) and three CAID patients homozygous for SGO1-K23E: M2 (SJi2551C2), M4 (SJi2690C2), and M5 (SJi2391C2). All participants gave written informed consent. The use of pluripotent stem cells was approved by the Research Ethics Committee of Sainte Justine University Hospital Center and the Ethics Committee of Montreal Heart Institute and carried out in accordance with guidelines from the Fonds de Recherche en Santé de Québec and the Declaration of Helsinki. The hiPSCs were generated from dermal fibroblasts at CHU Sainte Justine Stem Cell core using CytotuneTM 1.0 or 2.0 (Invitrogen) following manufacturer recommendation. The cell lines were then maintained in Essential 8^TM^ Flex medium on Matrigel^®^ (Corning) at 5% CO_2_ 37 °C incubator. The pluripotency was tested with RT-qPCR for marker genes using FastStart Universal SYBR Green (Roche). The primer sequences are listed in Supplementary Table 1. The lack of Sendai viral expression was tested with RT-qPCR before use in experiments. The cell lines were routinely checked for mycoplasma.

### Differentiation of hiPSC-CMs

A modified highly efficient differentiation method was used to produce beating monolayers^53^. Briefly, hiPSC colonies were passaged with 0.5 mM EDTA-PBS solution and seeded onto Matrigel-coated plates in E8 medium containing 5 µM Y27632 ROCK inhibitor (Selleckchem). The cells reached 70% cell confluence after 3 days in culture and were then treated with 5 µM CHIR99021 (Selleckchem) in RPMI1640 (Gibco) + B27 supplement for 1 day. On day 2, the cells were treated with 5 µM CHIR99021 + 10 ng recombinant human BMP-2 (Peprotech) + 1 µM SU5402 (Selleckchem) in RPMI1640 + B27 supplement. On day 3, the cells were treated with 10 µM IWP2 (Selleckchem) + 10 ng recombinant human BMP-2 + 1 µM SU5402 in RPMI1640 + B27 supplement. From day 4 to day 5 the cells were placed on the RPMI1640 + B27 supplement with 10 µM IWP2. On days 6 to11, the IWP2 was removed from the medium and the cells were cultured in Ham’s F12 medium + 5% KnockOut™ Serum Replacement (Thermo Fisher Scientific) with 2 ng FGF2 (Recombinant Human FGF-basic (146 a.a.), Peprotech). From day 12 onward, the cells were cultured in medium M199 + 5% Serum Replacement with 2 ng FGF2 and the media were refreshed every 2 days. To further purify the hiPSC-CMs for the Western blot and co-IP experiments, at day 21 the cells were starved for 3 days in glucose-free RPMI1640 + 5% Serum Replacement with 2 ng FGF2. Then the cells were placed on M199 + Serum Replacement with 2 ng FGF2 for 2 days and collected. For the patch-clamp experiments, the hiPSC-CMs were dissociated with TrypLE™ Select Enzyme (10X, Thermo Fisher Scientific) into single-cells, resuspended and replated on coverslips coated by Matrigel. After 2 days for recovery, the cells were utilized in patch-clamp experiments.

### Cell viability assay

PrestoBlue™ (Molecular Probes, Invitrogen) resazurin-based assay was utilized to determine NRVMs viability after the SGO1 knockdown 2 days. In total, 10 μL PrestoBlue reagent (/well) was added to 90 μL media for one well (96-well plate); the wells containing only media were used to correct for background color. After 30 min of incubation at 37 °C, 5% CO_2_, absorbance was measured using BioTek ELx800 Absorbance Microplate Reader (BioTek Instruments) at 570 nm and normalized to 600 nm values (reference wavelength). Absorbance readings were used to calculate for the cell viability for each group following manufacturer recommendation.

### Statistical analysis

Data are reported as the mean ± SEM. Multiple group statistical comparisons for I-V relations were obtained by 2-way ANOVA. Statistical analysis of the results of >2 groups was carried out by one-way ANOVA followed by the least significant difference test or Tukey test. Student’s *t* test was performed for comparison of 2 groups only. *P* < 0.05 was considered statistically significant; 2-tailed tests were used for all analyses. Analysis of the Western blot bands was performed with ImageJ 1.52 P image analysis software (NIH, USA). All measurements were obtained from distinct samples. Patch-clamp data were analyzed with Clampfit 9.0 (Axon, USA). GraphPad Prism 3.0 (GraphPad, CA) was used for other data analyses.

### Reporting summary

Further information on research design is available in the Nature Research Reporting Summary linked to this article.

## Supplementary information

Supplementary Information

Reporting Summary

## Data Availability

All data generated or analysed during this study are available within the Article and its Supplementary Information. All raw data supporting the findings from this study are available from the corresponding author upon reasonable request. Source data are provided with this paper.

## Source data

Source Data
