# Supplementary material for: Cohesin-protein Shugoshin-1 controls cardiac automaticity via HCN4 pacemaker channel
Source: Nat Commun. 2021 May 5;12:2551. doi: 10.1038/s41467-021-22737-5 (PMC8100125; doi:10.1038/s41467-021-22737-5)
Supplement: Supplementary file 1 — Supplementary Information [file 41467_2021_22737_MOESM1_ESM.pdf]

Supplementary Information for

Cohesin-protein shugoshin-1 controls cardiac automaticity via HCN4 pacemaker channel

Donghai Liu[1], Andrew Taehun Song[2,3], Xiaoyan Qi[1], Patrick Piet Van Vliet[3,4,6], Jiening Xiao[1], Feng Xiong[1,5], Gregor Andelfinger[3,7,8] & Stanley Nattel[1,5,9,10]*

[1]Montreal Heart Institute, Department of Medicine, Université de Montréal, Montréal, Québec, Canada.

[2]Department of Anatomy and Cell biology, McGill University, Montréal, Québec, Canada.

[3]Cardiovascular Genetics, Department of Pediatrics, Centre Hospitalier Universitaire Sainte-Justine Research Centre, University of Montreal, Montreal, Quebec, Canada.

[4]LIA (International Associated Laboratory) INSERM, Marseille, France.

[5]Department of Pharmacology and Therapeutics, McGill University, Montréal, Québec, Canada.

[6]LIA (International Associated Laboratory) Centre Hospitalier Universitaire Sainte-Justine, Montreal, Quebec, Canada.

[7]Department of Pediatrics, University of Montreal, Montreal, Quebec, Canada.

[8]Department of Biochemistry, University of Montreal, Montreal, Quebec, Canada.

[9]Institute of Pharmacology, West German Heart and Vascular Center, Faculty of Medicine, University Duisburg-Essen, Germany.

[10]IHU LIRYC Institute, Fondation Bordeaux Université, Bordeaux, France.

*Address correspondence to: Stanley Nattel, Montreal Heart Institute, 5000 rue Bélanger, Montréal H1T1C8, Québec, Canada. Phone: 514.376.3330; Email: stanley.nattel@icm-mhi.org

Items contained in this file:

Supplementary Figures 1-5

Supplementary Table 1

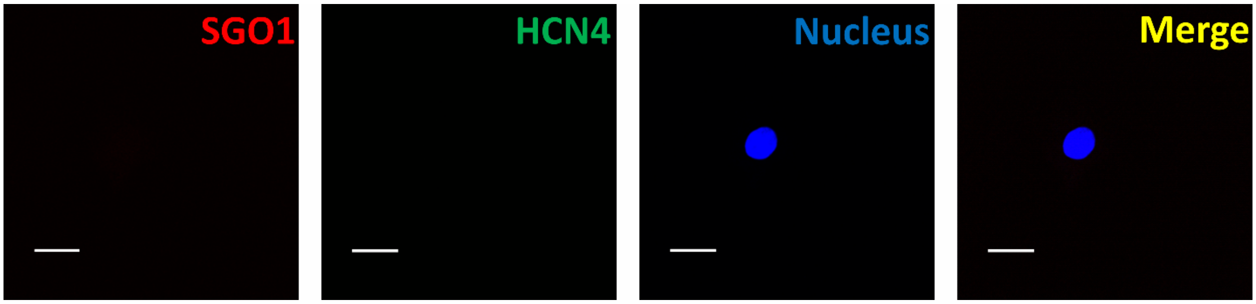

Supplementary Fig. 1 Specificity verification of HCN4 and SGO1 antibody for immunofluorescence (IF) experiments. Negative controls omitting the primary antibodies for SGO1 and HCN4 were used for IF in NRVMs. Scale bar=20 µm. This experiment was repeated 3 separate times.

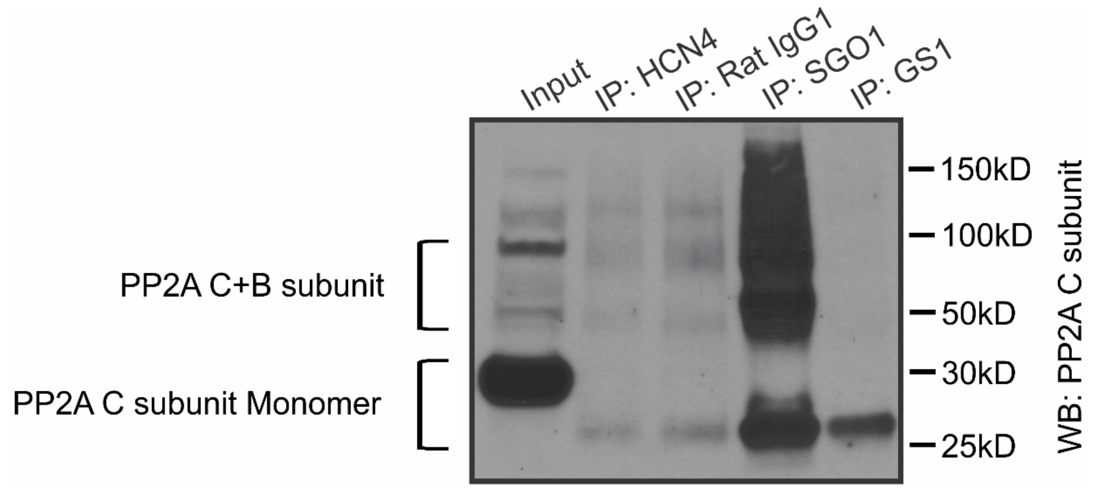

Supplementary Fig. 2 HCN4 does not interact with PP2a in NRVMs. HCN4 antibody or SGO1 antibody were used to pull down the protein complex in NRVMs lysates, PP2A C subunit antibody was used to detect PP2A C subunit bands. Rat IgG1 or glycogen synthase 1 (GS1) antibody were used as the isotype antibody control for HCN4 antibody or SGO1 antibody. NRVMs lysates were loaded as the input control. IP: immunoprecipitation. WB: western blot. Similar results were obtained in *n*=3 biologically independent experiments.

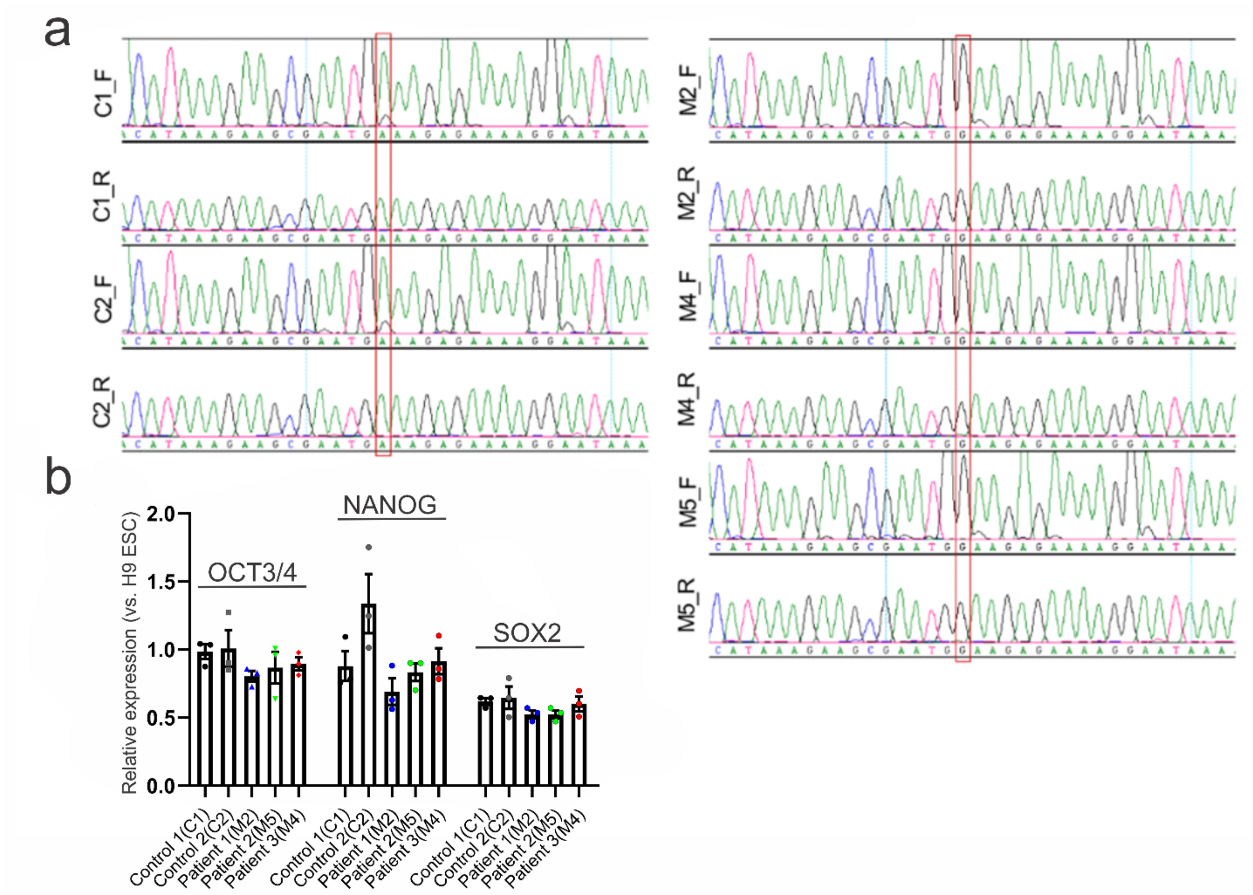

Supplementary Fig. 3 Generation of hiPSC lines. a, Sanger sequencing result for hiPSC lines. Red boxes show the mutated site c.69A>G in patient-specific hiPSC line. **b**, Quantitative PCR for pluripotent marker genes in hiPSC lines. Normalized with GAPDH expression and fold change in comparison to H9 embryonic stem cell. Data are mean ± SEM, *n*= 3 biologically independent experiments.

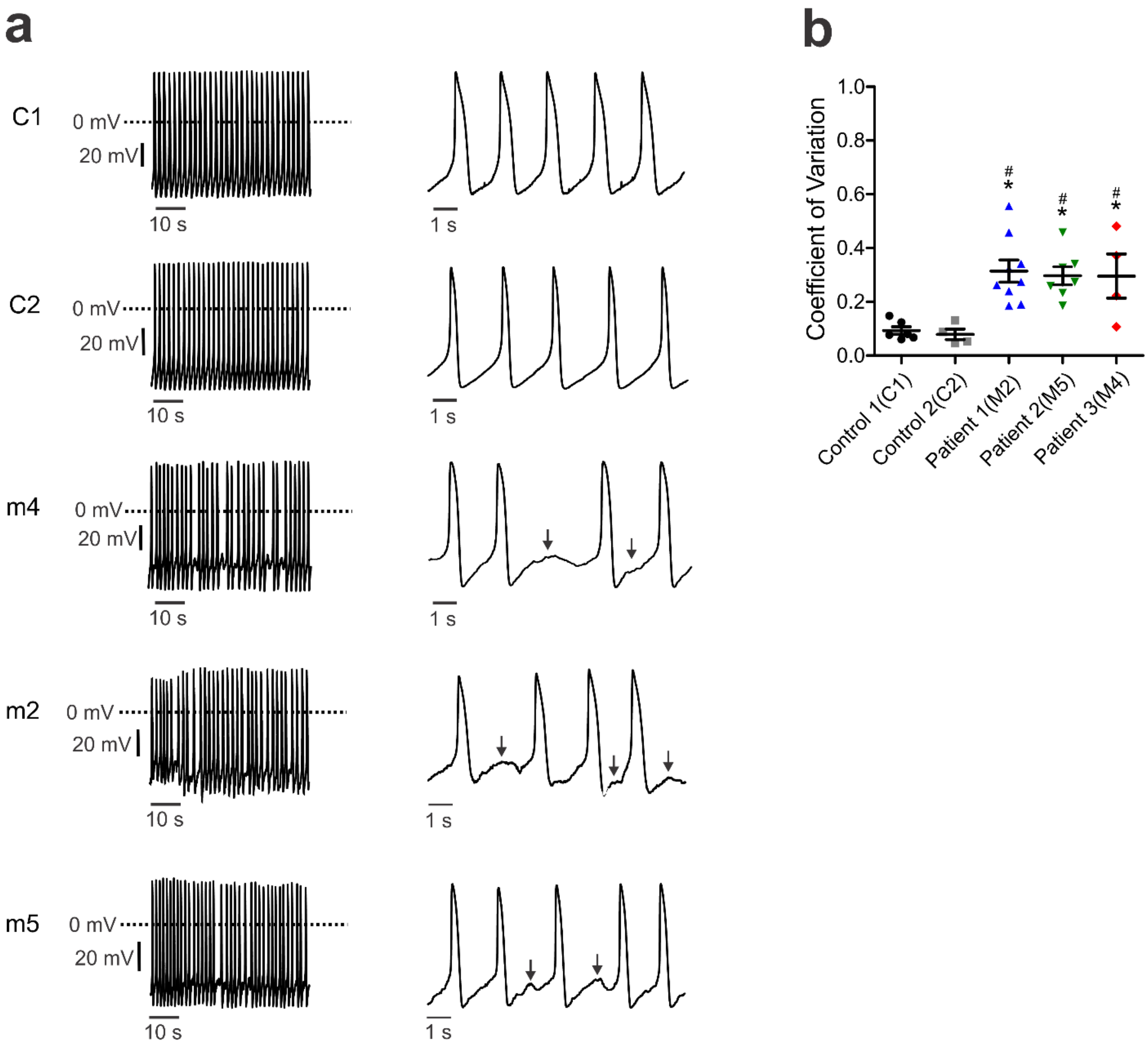

Supplementary Fig. 4 Spontaneous action potentials in single hiPSC-CMs. a, Representative recordings of atrial-like action potentials in healthy control and patient-specific iPSC-CMs. **b**, Analysis of coefficient of variation of atrial-like action potential firing in healthy control and CAID patient-specific hiPSC-CMs. n_{C1}=6, n_{C2}=6, n_{M2}=9, n_{M5}=7 and n_{M4}=4 biologically independent cells. Data are expressed as mean ± SEM. One-way ANOVA with Tukey's multiple comparison test. **P < 0.05 vs* C1, $^{\#}$*P < 0.05 vs* C2.

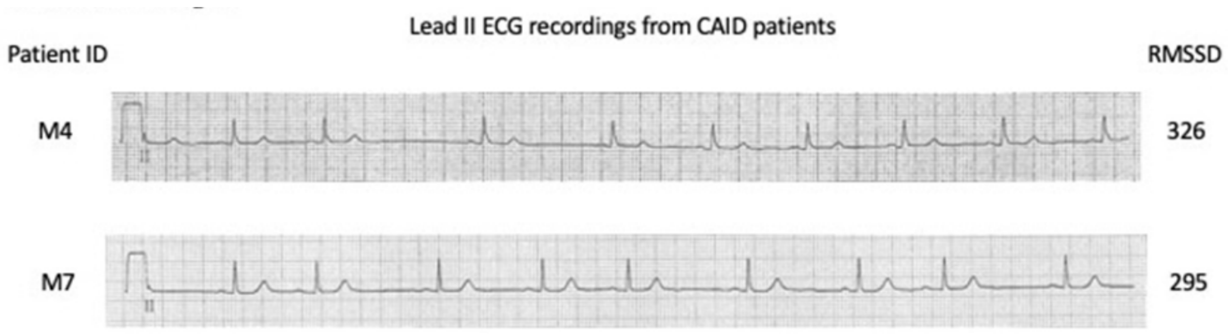

Supplementary Fig. 5 Electrocardiogram lead II rhythm strips from 2 CAID patients. RMSDD= root mean square of successive differences.

Supplementary Table 1. The primers used in this study.

Gene	catalog number (company) / Sequence
Rat HCN1	Rn00670384_m1 (Thermo Fisher Scientific)
Rat HCN2	Rn01408572_mH (Thermo Fisher Scientific)
Rat HCN3	Rn00586666_m1 (Thermo Fisher Scientific)
Rat HCN4	Rn00572232_m1 (Thermo Fisher Scientific)
Rat GAPDH	Rn01775763_g1 (Thermo Fisher Scientific)
Human GAPDH (forward)	GAAGGTGAAGGTCGGAGT
Human GAPDH (reverse)	GAAGATGGTGATGGGATTTC
Human OCT3/4 (forward)	AGTGAGAGGCAACCTGGAGA
Human OCT3/4 (reverse)	ACACTCGGACCACATCCTTC
Human NANOG (forward)	GGATCCAGCTTGTCCCCAAAGCTTG
Human NANOG (reverse)	CTGGAGGCTGAGGTATTTCTGTCTC
Human SOX2 (forward)	GACAGTTACGCGCACATGAA
Human SOX2 (reverse)	AGCCGTTCATGTAGGTC
