# Supplementary material for: Cohesin-protein Shugoshin-1 controls cardiac automaticity via HCN4 pacemaker channel
Source: Nat Commun. 2021 May 5;12:2551. doi: 10.1038/s41467-021-22737-5 (PMC8100125; doi:10.1038/s41467-021-22737-5)
Supplement: Supplementary file 2 — Reporting Summary [file 41467_2021_22737_MOESM2_ESM.pdf]

nature research

Corresponding author(s): Stanley Nattel

Last updated by author(s): Feb 16, 2021

Reporting Summary

Nature Research wishes to improve the reproducibility of the work that we publish. This form provides structure for consistency and transparency in reporting. For further information on Nature Research policies, see our Editorial Policies and the Editorial Policy Checklist.

Statistics

For all statistical analyses, confirm that the following items are present in the figure legend, table legend, main text, or Methods section.

n/a	Confirmed	
☐	☒	The exact sample size (*n*) for each experimental group/condition, given as a discrete number and unit of measurement
☐	☒	A statement on whether measurements were taken from distinct samples or whether the same sample was measured repeatedly
☐	☒	The statistical test(s) used AND whether they are one- or two-sided<br>*Only common tests should be described solely by name; describe more complex techniques in the Methods section.*
☐	☒	A description of all covariates tested
☐	☒	A description of any assumptions or corrections, such as tests of normality and adjustment for multiple comparisons
☐	☒	A full description of the statistical parameters including central tendency (e.g. means) or other basic estimates (e.g. regression coefficient) AND variation (e.g. standard deviation) or associated estimates of uncertainty (e.g. confidence intervals)
☐	☒	For null hypothesis testing, the test statistic (e.g. *F*, *t*, *r*) with confidence intervals, effect sizes, degrees of freedom and *P* value noted<br>*Give P values as exact values whenever suitable.*
☒	☐	For Bayesian analysis, information on the choice of priors and Markov chain Monte Carlo settings
☒	☐	For hierarchical and complex designs, identification of the appropriate level for tests and full reporting of outcomes
☒	☐	Estimates of effect sizes (e.g. Cohen's *d*, Pearson's *r*), indicating how they were calculated

Our web collection on statistics for biologists contains articles on many of the points above.

Software and code

Policy information about availability of computer code

Data collection	ZEN 2.6 was used for confocal images collection, Clampex 10.4 was used for patch-clamp data recording.
Data analysis	Analysis of the Western blot bands was performed with ImageJ image 1.52p (NIH, USA). Patch-clamp data were analyzed with Clampfit 9.0 (Axon, USA). GraphPad Prism 3.0 (GraphPad, CA) was used for other data analyses

For manuscripts utilizing custom algorithms or software that are central to the research but not yet described in published literature, software must be made available to editors and reviewers. We strongly encourage code deposition in a community repository (e.g. GitHub). See the Nature Research guidelines for submitting code & software for further information.

Data

Policy information about availability of data

All manuscripts must include a data availability statement. This statement should provide the following information, where applicable:

- Accession codes, unique identifiers, or web links for publicly available datasets
- A list of figures that have associated raw data
- A description of any restrictions on data availability

All data generated or analysed during this study are available within the Article and its Supplementary Information. All source data for graphs included in the paper are provided in the source data Excel file. All raw data supporting the findings from this study are available from the corresponding author upon reasonable request, as indicated in the manuscript lines 825-826.

Field-specific reporting

Please select the one below that is the best fit for your research. If you are not sure, read the appropriate sections before making your selection.

- [x] Life sciences
- [ ] Behavioural & social sciences
- [ ] Ecological, evolutionary & environmental sciences

For a reference copy of the document with all sections, see nature.com/documents/nr-reporting-summary-flat.pdf

Life sciences study design

All studies must disclose on these points even when the disclosure is negative.

Sample size	Sample sizes were determined based on our extensive past experience with these types of experiments (PMID: 28188930; 29574723; 26139058) and the studies from other groups (PMID: 31114854). All experiments were conducted with at least three independent experiments and multiple independent biological replicates. For electrophysiology experiments comparing multiple cells for different cell-lines or groups, n>10 was required and we generally aimed for Ns of 15-40 to control adequately for inter-cell variability, following our experience and prior reports in the literature. For biochemical analyses, we required at least 4-6 independent experiments with each group. Adequacy of sample size was confirmed on the basis that statistically non-significant differences were of an order that suggested lack of biological significance.
Data exclusions	On some occasions, no spontaneous action potentials were observed in healthy control and patient iPSC-CMs. There was one cell without meaningful action potentials in control 1 (C1), one cell in patient 1(M2) and 1 cell in patient 2(M5); these were not analyzable for frequency and were not included in analyses (Supplementary Fig. 7c). This information has been added to the relevant Figure Legend. There were no exclusions for other experiments.
Replication	All experimental series were repeated in different experiments and separate days. Each N in the paper represents a measurement on a separate cell or preparation; repeated measurements were not obtained. Replication was ensured by performing a sufficient number of independent measurements, each on separate samples or subjects (cells), to ensure replication of the biological phenomenon being measured.
Randomization	Randomization was not possible in the studies on hiPSC samples because the sample identity was determined by its source. For the studies involving interventions, no formal randomization was performed but group allocation was arbitrary and not related to any properties of samples or subjects.
Blinding	Investigators were not blinded to group allocation. This work spanned over 6 years and at the outset of the studies, blinding was not commonly performed in the lab and was not stressed in the experimental literature. It made no sense to change the practice towards the end of the study when this practice of blinding became more common. We are now routinely performing studies blinded in the lab. No in vivo experiments were conducted in this study.

Reporting for specific materials, systems and methods

We require information from authors about some types of materials, experimental systems and methods used in many studies. Here, indicate whether each material, system or method listed is relevant to your study. If you are not sure if a list item applies to your research, read the appropriate section before selecting a response.

Materials & experimental systems

n/a	Involved in the study	
[ ]	[x]	Antibodies
[x]	[ ]	Eukaryotic cell lines
[x]	[ ]	Palaeontology and archaeology
[ ]	[x]	Animals and other organisms
[ ]	[x]	Human research participants
[x]	[ ]	Clinical data
[x]	[ ]	Dual use research of concern

Methods

n/a	Involved in the study	
[x]	[ ]	ChIP-seq
[x]	[ ]	Flow cytometry
[x]	[ ]	MRI-based neuroimaging

Antibodies

Antibodies used	Certified and company-validated commercial antibodies were used in this study:<br>SGO1 1:1000 dilution for WB, 1:200 dilution for IF, 2ug/sample for co-IP, Abcam, ab58023, mouse monoclonal antibody (Lot#GR3297345-1).<br>HCN4 1:1000 dilution for WB, 1:200 dilution for IF, Alomone Labs, APC-052, 4ug/sample for co-IP, rabbit polyclonal antibody (Lot#APC052AN2402).<br>HCN2 1:1000 dilution for WB, Alomone Labs, APC-030, rabbit polyclonal antibody (Lot#APC030AN1102).<br>GAPDH 1:10000 dilution for WB, Fitzgerald, 10R-G109a, mouse monoclonal antibody (Lot#GR3297345-1, clone: 6c5).<br>GFP 1: 1000 dilution for WB, Invitrogen, MA5-15256, mouse monoclonal antibody (Lot#2916, clone: GF28R).

mCherry 1: 1000 dilution for WB, invitrogen, 4 ug/sample for co-IP, Invitrogen, M11217, rat monoclonal antibody (Lot# TL276838, clone: 16D7).
Phospholamban 4ug/sample for co-IP, Thermo Fisher Scientific, MA3-922, mouse monoclonal antibody (Lot#092-12, clone 2D12).
PP2A C subunit 1: 1000 dilution for WB, Sigma-Aldrich, SAB4200266, mouse monoclonal antibody (Lot#021M4767, clone: 7A6).
glycogen synthase1 4ug/sample for co-IP, Sigma-Aldrich, SAB4200266, mouse monoclonal antibody (Lot#021M4767, clone: 7A6).
Donkey anti rabbit secondary antibody 1:5000 dilution, Jackson, #711035152; Donkey polyclonal antibody (Lot# 126333)
Donkey anti mouse secondary antibody 1:5000 dilution, Jackson, #711035151; Donkey polyclonal antibody (Lot#125459)
Donkey anti rat secondary antibody 1:5000 dilution, Invitrogen, #A18745, Donkey polyclonal antibody (Lot# 6850020510)

Validation

All antibodies have been validated by the companies from which they were purchased. The subcellular localization of HCN4 and SGOL1 antibody is consistent with published literature in this field. Details about validation statements of the manufacturer, relevant citations and antibody profiles can be found on manufacturer's website.

SGOL1 (ab58023): https://www.abcam.com/shugoshin-antibody-ab58023.html
SGOL1 (ab58023) antibody was validated by the manufacturer using Hela cells, MCF-7 cells and recombinant protein for western blot or immunofluorescence. We verified that this antibody for immunohistochemistry (PMID 28465207). We verified that this antibody stains the cell nucleus, cytosol and membrane as expected. We verified the antibody for Western blot with overexpression of SGO1 and knockdown of SGO1 in neonatal rat ventricular myocytes, changes inidentified bands were as expected. We verified the antibody for co-immunoprecipitation experiments in neonatal rat ventricular myocytes, as expected.
More than 11 citations.

HCN4 (apc-052): https://www.alomone.com/p/anti-hcn4-2/APC-052
HCN4 (apc-052) antibody was validated by the manufacturer using HCN4 knockout mice heart lysate for western blot, rat thalamus for co-immunoprecipitation and mouse sinoatrial myocytes for immunofluorescence.We verified the antibody for western blot and co-immunoprecipitation in neonatal rat ventricular myocytes, as expected.We verified that this antibody stains the nuclei in neonatal rat ventricular
myocytes, as expected. More than 27 citations.

HCN2 (apc-030): https://www.alomone.com/p/anti-hcn2/APC-030
HCN2 (apc-030) antibody was validated by the manufacturer using rat brain lysate for western blot, rat thalamus lysate for co-immunoprecipitation and HCN2 knockout mice for immunofluorescence. We verified this antibody in neonatal rat ventricular myocytes for Western blot, as expected. More than 20 citations.

GAPDH (10R-G109a): https://www.fitzgerald-fii.com/gapdh-antibody-10r-g109a.html
GAPDH (10R-G109a) antibody was validated by manufacturer for ELISA, immunofluorescence and western blot. We verified this antibody in neonatal rat ventricular myocytes for western blot, as expected. More than 182 citations.

GFP (MA5-15256): https://www.thermofisher.com/antibody/product/GFP-Antibody-clone-GF28R-Monoclonal/MA5-15256
GFP (MA5-15256) antibody was validated by manufacturer for ELISA, immunofluorescence, immunoprecipitation and western blot in GFP-transfected HeLa cells and HEK293 cells. We verified this antibody in neonatal rat ventricular myocytes for western blot with overexpression. More than 43 citations.

mCherry (M11217): https://www.thermofisher.com/antibody/product/mCherry-Antibody-clone-16D7-Monoclonal/M11217
mCherry (M11217) antibody was validated by manufacturer for flow cytometry , immunocytochemistry, immunofluorescence, immunohistochemistry, immunoprecipitation and western blot in U2OS cells, Hela cells and transgenic mice expressing mCherry. We verified this antibody in neonatal rat ventricular myocytes for western blot with overexpression and co-immunoprecipitation experiments. More than 58 citations.

Phospholaman (MA3-922): https://www.thermofisher.com/antibody/product/Phospholamban-Antibody-clone-2D12-Monoclonal/MA3-922
Phospholamban (MA3-922) antibody was validated by manufacturer for immunocytochemistry, immunofluorescence, immunohistochemistry, immunoprecipitation and western blot in C2C12 cells, mouse heart and human heart. We verified this antibody in neonatal rat ventricular myocytes for co-immunoprecipitation as a negative control, the result is negative as expected. More than 138 citations.

PP2A C subunit (SAB4200266): https://www.sigmaaldrich.com/catalog/product/sigma/sab4200266?lang=en&region=CA
PP2A C subunit (SAB4200266) antibody was validated by manufacturer for immunoprecipitation and western blot in RAT2 cells, NTH-3T3 cells and A431 cells. We verified this antibody in neonatal rat ventricular myocytes for co-immunoprecipitation. More than 8 citations.

glycogen synthase1 (sc-81173): https://www.scbt.com/p/glycogen-synthase-1-antibody-gs-7h5
glycogen synthase1 (sc-81173) antibody was validated by manufacturer for immunoprecipitation, Western blot and immunofluorescence in rabbit and mouse skeletal muscle. We verified this antibody in neonatal rat ventricular myocytes for co-immunoprecipitation as a negative control, the result is negative as expected. More than 7 citations.

Animals and other organisms

Policy information about studies involving animals; ARRIVE guidelines recommended for reporting animal research

Laboratory animals	Neonatal rat pups of both sexes were used and we did not determine the rat pup sex. The estimated sex ratio was 1:1. They were obtained from pregnant female Wistar rats and studied at 1-2 days of age.
Wild animals	This study did not involve wild animals
Field-collected samples	This study did not involve samples collected from the field.
Ethics oversight	Montreal Heart Institute Animal Research Ethics Committee.

Note that full information on the approval of the study protocol must also be provided in the manuscript.

Human research participants

Policy information about studies involving human research participants

Population characteristics	The donor of healthy control 1(C1) iPSC line, male, 40 years old.<br>The donor of healthy control 2(C2) iPSC line, male, 41 years old.<br>The donor of CAID patient 1(M2) iPSC line, male, 27 years old.<br>The donor of CAID patient 2(M5) iPSC line, female, 48 years old.<br>The donor of CAID patient 3(M4) iPSC line, male, 21 years old.<br>Control cell lines were confirmed to be homozygous wildtype for the SGO1 allele. Cell lines from CAID patients were confirmed to be homozygous for the SGO1 K23E mutation. For comparison, see supplementary Fig. 6a, which was generated using bidirectional Sanger sequencing.
Recruitment	Control cell lines were obtained from donors who self-referred to our institutions. CAID cell lines were obtained from patients recruited within a biobank located in CHU Sainte Justine. We are not aware of any biases that could have affected our resultse
Ethics oversight	The use of induced pluripotent stem cells was approved by the Research Ethics Committee of Sainte Justine University Hospital Center and the Ethics Committee of Montreal Heart Institute.

Note that full information on the approval of the study protocol must also be provided in the manuscript.
